# DeepLocRNA: an interpretable deep learning model for predicting RNA subcellular localization with domain-specific transfer-learning

**DOI:** 10.1093/bioinformatics/btae065

**Published:** 2024-02-05

**Authors:** Jun Wang, Marc Horlacher, Lixin Cheng, Ole Winther

**Affiliations:** Bioinformatics Centre, Department of Biology, University of Copenhagen, København Ø 2100, Denmark; Computational Health Center, Helmholtz Center Munich, Neuherberg 85764, Germany; Shenzhen People’s Hospital, First Affiliated Hospital of Southern University of Science and Technology, Second Clinical Medicine College of Jinan University, Shenzhen 518020, China; Bioinformatics Centre, Department of Biology, University of Copenhagen, København Ø 2100, Denmark; Center for Genomic Medicine, Rigshospitalet (Copenhagen University Hospital), Copenhagen 2100, Denmark; Section for Cognitive Systems, Department of Applied Mathematics and Computer Science, Technical University of Denmark, Kongens Lyngby 2800, Denmark

## Abstract

**Motivation:**

Accurate prediction of RNA subcellular localization plays an important role in understanding cellular processes and functions. Although post-transcriptional processes are governed by trans-acting RNA binding proteins (RBPs) through interaction with cis-regulatory RNA motifs, current methods do not incorporate RBP-binding information.

**Results:**

In this article, we propose DeepLocRNA, an interpretable deep-learning model that leverages a pre-trained multi-task RBP-binding prediction model to predict the subcellular localization of RNA molecules via fine-tuning. We constructed DeepLocRNA using a comprehensive dataset with variant RNA types and evaluated it on the held-out dataset. Our model achieved state-of-the-art performance in predicting RNA subcellular localization in mRNA and miRNA. It has also demonstrated great generalization capabilities, performing well on both human and mouse RNA. Additionally, a motif analysis was performed to enhance the interpretability of the model, highlighting signal factors that contributed to the predictions. The proposed model provides general and powerful prediction abilities for different RNA types and species, offering valuable insights into the localization patterns of RNA molecules and contributing to our understanding of cellular processes at the molecular level. A user-friendly web server is available at: https://biolib.com/KU/DeepLocRNA/.

## Graphical Abstract

**Graphical Abstract:**
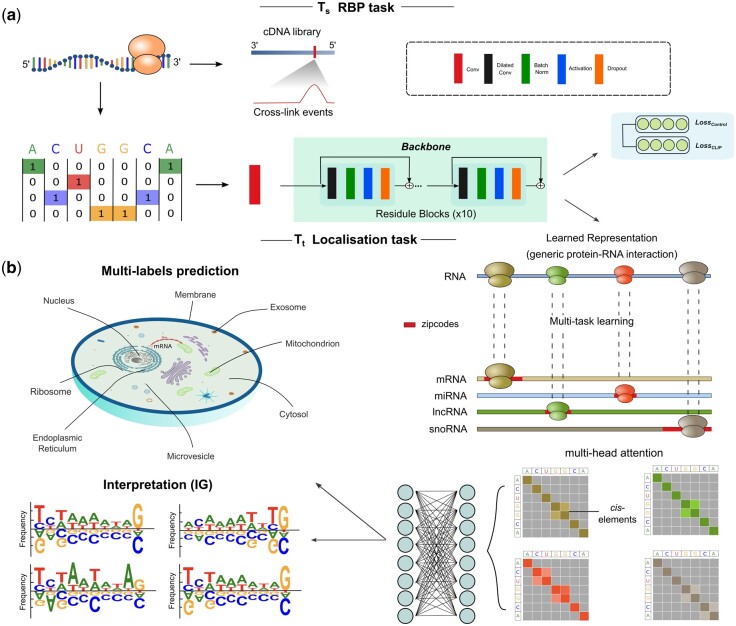

## 1 Introduction

RNA localization is the process of transporting and anchoring RNA molecules to specific subcellular regions, where they can perform their functions in gene expression, cell differentiation, and development (Jansova *et al.* 2018, Engel *et al.* 2020, Das *et al.* 2021, Wang *et al.* 2023). The mis-regulation and perturbation of RNA localization are relevant to various disease phenotypes, including cancer (Leucci *et al.* 2016, Neelamraju *et al.* 2018, Panda *et al.* 2018, Zhang *et al.* 2020), development disorders (Nousiainen *et al.* 2008, Batista *et al.* 2011, Jao *et al.* 2017, Okamura *et al.* 2019), and disorders involving neuromuscular or neuronal dysfunction (Bassell and Warren 2008, Dictenberg *et al.* 2008, Ivy *et al.* 2010, Baleriola *et al.* 2014, Didiot *et al.* 2018). To play a role in cellular regulation, RNA molecules are transported from the nucleus to target compartments and regulated by RNA binding proteins through three primary mechanisms: (i) direct transport, (ii) protection from mRNA degradation, and (iii) diffusion and local entrapment (Das *et al.* 2021). All these localization mechanisms require coupled protein components to interact with the RNAs to form a ribonucleoprotein (RNP) complex. This essential interaction is primarily driven by *cis-*regulatory elements, also known as zip codes, which serve as key factors in the linear RNA sequence or structure. They determine the interaction between RNAs and the RNA binding domains (RBDs) of RNA binding proteins (RBPs) (Hafner *et al.* 2021), directing RNA to designated organelles.

Characterizing the factors involved in an RNP complex is important for understanding how RNA traffics from its nascent state in the nucleus to regions outside the nucleus. Cross-linking and immunoprecipitation followed by sequencing (CLIP-seq) is the most common protein-centric experimental approach to measure the protein-RNA interaction profile across the whole transcriptome. Specifically, the method employs UV light to create an irreversible covalent bond between proteins and RNA in their immediate vicinity. This is done before immunoprecipitation purification, protein digestion, cDNA library sequencing and bioinformatics analysis (Hafner *et al.* 2021). There are several variants of CLIP, such as individual-nucleotide resolution CLIP (iCLIP) (König *et al.* 2010), enhanced CLIP (eCLIP) (Van Nostrand *et al.* 2016), and m6A individual-nucleotide resolution UV crosslinking and immunoprecipitation (miCLIP) (Linder *et al.* 2015), which have different modifications in their purification and cDNA library preparation, enabling the protection of the truncations in the protein-RNA interaction sites that helps to increase the specificity and reach to the single nucleotide resolution of the RNA–protein interaction detection.

Currently, there are several machine learning-based tools available for predicting the localization of transcripts. These tools can be broadly categorized into two main types—image-based and sequence-based models. Image-based models leverage manually curated features to characterize RNA distributions (Yan *et al.* 2019, Garg *et al.* 2020) or employ cutting-edge computer vision methods to learn hidden feature representation (Glisovic *et al.* 2008). Sequence-based models (Garg *et al.* 2020, Wang *et al.* 2021, Wang *et al.* 2023) predict the localization derived from the primary sequence. The inherent features of *cis-*regulatory elements and the secondary structure are biologically relevant for determining where transcripts should be transported through binding with RBPs. However, predicting localization exclusively based on the primary sequence may have inherent defects as the primary sequences themselves do not contain RBP binding information. A single sequence can bind with different RBPs, indicating that the regulation of RNA trafficking is a sophisticated and systematic RNA–protein binding network (Clouse *et al.* 2008). Ideally, measuring transcriptome-wide RNA–protein interactions would deliver a broad interaction profile between RBPs and RNAs, revealing the numerous regulatory aspects of co- and post-transcriptional gene expression, including RNA splicing, polyadenylation, capping, modification, export, localization, translation and turnover (Keene 2007, Glisovic *et al.* 2008).

In this study, we propose DeepLocRNA, an RNA localization prediction tool based on fine-tuning a multi-task RBP-binding prediction model, which was pre-trained to predict the signal of a large cohort of eCLIP data at single nucleotide resolution. We demonstrate that our model can gain performance from the learned RBP binding information to downstream localization prediction across four RNA species and perform robustly to predict the localization with a limited training dataset. Furthermore, we also apply our model on training in multiple species data and extend the application in a biologically interpretable manner. A user-friendly web server is available at: https://biolib.com/KU/DeepLocRNA/.

## 2 Materials and methods

### 2.1 Localization data source

The localization data used in this study were initially collected from the RNALocate2.0 database (Cui *et al.* 2022) (http://www.rna-society.org/rnalocate1/), which provides the annotated RNA localization information supported by experimental evidence. Then, we retrieve the paired RNA sequences from different sources (Supplementary Text). A unified benchmarking dataset was built for humans and mice, including mRNA, lncRNA, miRNA, and snoRNA. To prevent data leakage, we employed CD-HIT-EST (Fu *et al.* 2012) to eliminate redundant sequences, resulting in Nucleus (13 352), Exosome (22 335), Cytosol (2587), Cytoplasm (10 026), Ribosome (5226), Membrane (3356), ER (1977), Microvesicle (1958), and Mitochondrion (33) (Supplementary Table S4). Curated datasets were split into 5-fold subsets according to the RNA types and the distribution of the constitution of localization. For example, genes with labels as “111000000,” which means they have the label of Nucleus, Exosome, and Cytosol, will be split accordingly in mRNA and miRNA if they exist in these two RNA species. Otherwise, only one of them will take each fold. To compare with other counterparts, the independent benchmarking dataset was held out from the unified benchmarking dataset. Mouse sequence data were processed the same as it was implemented in the human unified dataset, including reducing the redundant sequences and train test split (Supplementary Text).

### 2.2 Model structure

The architecture of DeepLocRNA is provided in Supplementary Fig. S6. It is an end-to-end differentiable model that consists of a pre-trained RBP sequence-to-signal encoder (Supplementary Text), followed by an attention block and ending in a multi-class classification head. RBP binding signals are extracted to supervise the CNN to focus not only on the sequence composition but also on the RBP potential binding signals. After the RBP-aware encoding, a self-attention layer is applied to allow the model to extract information from relevant parts of the sequence (Bahdanau *et al.* 2014) (Supplementary Text). The attention layer maps from sequence to a fixed-length representation that is then fed into a simple fully connected classification network.

### 2.3 Model training

To minimize the difference between true multilabel and predicted probabilities, we employed a binary cross-entropy loss function tailored for multilabel classification tasks.

As the data from each class have clear imbalance issues, we took class weights into account to address data imbalance challenges. The weighting scheme in the loss function was formulated as follows:

(1) $${\mathrm{Loss}}_{j}=-\sum_{j=1}^{m}W_{j}[y_{i,j}\;\log(p_{i,j})+(1-y_{i,j})\log(1-p_{i,j})],$$

(2) $$\;W_{j}=\;\frac{\sum_{i=1}^{n}y_{i,j}}{\sum_{i=1}^{n}\sum_{j=1}^{m}y_{i,j}},$$

where $y_{i,j}\;\in \;\{0,\;1\}$ is the true label and $p_{ij}\;\in \;[0,\;1]$ denote the predicted probability values of the model. There are variant labels in different training schemes. For example, we utilized seven-compartment labels while training the mRNA model. Hence, each $y_{j}$ indicates 7 labels in m ∈ {1, 2, …, 7}. Furthermore, the weight of each class $W_{j}$ was defined by the proportion of sample size in each class accordingly. Because of the label inconsistency, we exempt the weights to calculate the loss function when training the unified model.

To make the training process stable, the gradient clip was applied to prevent gradient-related challenges (Supplementary Test). Adam stochastic optimization method with a learning rate of 0.005 and set the weight decay as 1e−5 to prevent overfitting. To discern distinct RNA types when training the unified model, we incorporate identity tags with four dimensions within the fully connected layer.

The entire model was trained based on the PyTorch deep learning framework, and PyTorch-lightning, a lightweight PyTorch wrapper, was implemented to simplify the process of organizing and training the PyTorch model. PyTorch-lightning streamlined the training workflows, automating tedious tasks such as setting up training loops, handling device placement on 4 NVIDIA A100 GPUs with 40 GB memory underlining the Distributed Data Parallel strategy, which keeps repeats of the model in different GPUs and split the data while training synchronously. This not only saved valuable development time but also ensured the efficient utilization of powerful hardware resources.

### 2.4 Model evaluation

In our model evaluation, we employed a comprehensive assessment approach, focusing on four key performance metrics: F1 score, Matthews Correlation Coefficient (MCC), Area Under the Receiver Operating Characteristic curve (AUROC), and Area Under the Precision-Recall curve (AUPRC). The AUROC and AUPRC were specifically utilized to gauge the model’s robustness, and the F1 score and MCC were employed to evaluate the model’s statistical accuracy. All gathered RNA sequences (see Supplementary Table S4) were incorporated during the training phase, while infrequent data with a size <40 were excluded during the evaluation process.

To determine these optimal thresholds when calculating MCC and F1 scores, we leveraged the test dataset to identify the threshold for each RNA compartment that yielded the highest MCC. Separate thresholds were established for various RNA types, with these final thresholds subsequently applied to the predictive server (Supplementary Figs S8–S11) For instance, in the context of mRNA classification, 0.7551 for the nucleus, 0.9796 for exosome, 0.2245 for cytosol, 0.2857 for ribosome, 0.3061 for membrane, and 0.1837 for the ER.

### 2.5 Model explanation

To provide a clearer illustration of the attention mechanism, the attention weights serve to showcase how the model dynamically directs its focus onto the sequence. Given that most *cis-*regulatory elements were predominantly found at two ends of the sequence, we selectively truncated the sequence to keep these critical regions. Specifically, we focused our analysis on mRNA sequences to maintain both the 5ʹUTR and the 3ʹUTR. Sequences exceeding 2000 nt were selected, and 2000 nt were trimmed from two ends to establish a uniform sequence length. For the computation of attention weights, we calculated *z*-scores across attention heads and determined a mean value over the pooled sequence length of 1000. Subsequently, we applied min–max normalization to standardize the attention weights within a range of 0–1 for enhancing visualization. To restore the full length we simply replicated the pooled sequences 8 times to get back to 8000 nt.

We used the Integrated Gradients (IG) (Sundararajan *et al.* 2017) to extract critical motifs with a high level of informativeness, essential for RNA localization prediction. To enhance our analysis, we divided the dataset into eight distinct compartments, allowing us to pinpoint the most frequently occurring and influential motifs within each compartment. The overall IG scores were computed using sequences truncated to 2500 nucleotides from both the 5ʹ and 3ʹ ends. Subsequently, we aggregated attribution scores for each position within the sequence across four nucleotide dimensions. We identified 5-mer motifs by sliding a 5-nucleotide window across the 8000-sequence length, selecting the 5-mer with the highest IG score for each sequence. Next, we pinpointed the top 5 maximum attribution values within each compartment dataset, representing the most impactful motifs driving sequence trafficking. Finally, these top 5 effective motifs for each compartment were compared with the top 2 motifs extracted from the RBPnet dataset (Horlacher *et al.* 2023).

### 2.6 DeepLocRNA webserver

We provide a user-friendly web server, https://biolib.com/KU/DeepLocRNA/, powered by the Biolib library that has been developed to provide secure access to bioinformatics tools directly within the browser. Users can obtain predicted localization results by uploading a FASTA-formatted file or downloading the locally installable version of DeepLocRNA. The server supports optional specification of species and RNA types for running the prediction model.

## 3 Results

### 3.1 Benchmarking with the other tools

**Figure 1.**
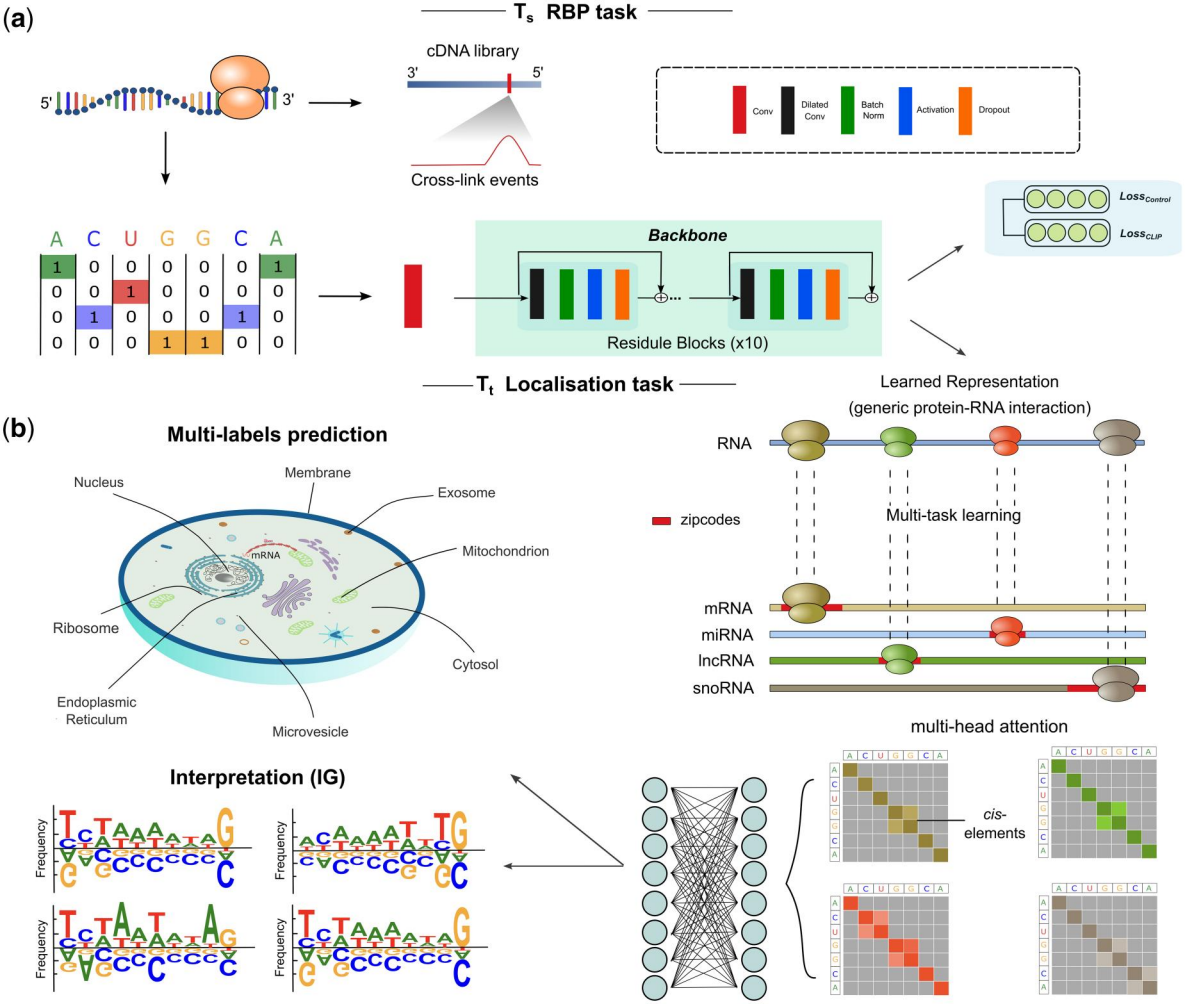
A comprehensive visualization of the pre-training and fine-tuning schemes used in localization prediction. (**a**) Sequences are one-hot encoded before serving as the input to our RBP sequence-to-signal model, which enables the prediction of the RBP binding signal in a single nucleotide resolution. After ten rounds of feature extraction, the final feature embeddings were generated to yield the representation of protein-RNA interaction, getting the Ts pre-trained backbone model. (**b**) RBP binding signals are used to guide the localisation prediction across 8 compartments Tt. Before going to the fully connected layer, the multi-head self-attention mechanism is used to attend the cis-regulatory zipcodes. When the multi-label localisation results are predicted, functional motifs can be extracted to do the model interpretation derived from the IG score across 4 nucleotide dimensions.

Our model construction initiates with a pre-trained backbone model predicting RBP-binding profiles for eCLIP datasets from ENCODE database (Van Nostrand *et al.* 2020) (Section 2). Then, the backbone model was fine-tuned using a diverse set of RNA localization data (lncRNA, miRNA, snoRNA, mRNA) (Fig. 1). A fine-tuned model was employed for benchmarking against counterparts trained on a specific RNA type.

We divided the mRNA dataset from our unified benchmarking dataset to ensure a fair comparison with three other predictive tools: DM3Loc (Wang *et al.* 2021), iLocmRNA (Zhang *et al.* 2021), and mRNALoc (Garg *et al.* 2020). The results unequivocally demonstrate that fine-tuned DeepLocRNA outperforms the other three methods in terms of overall performance across six compartments with the highest macro AUROC of 0.7493 and the best AUROC in 5 of 6 compartments (Table 1, Supplementary Table S1). When training the model from scratch, a lower AUROC of 0.7283 was obtained (Table 1). In comparison to DM3Loc, our model exhibits considerable advancements in Exosome localization prediction (AUROC from 0.7273 to 0.7633, Supplementary Table S1). We achieved higher performance by assigning weights to the loss function based on the abundance of each compartment (Supplementary Table S1). The training strategy employing early stopping also illustrates a more rapid descent in loss and a lower final loss value when compared with DM3Loc (Supplementary Fig. S2).

**Table 1.** The average performance of DeepLocRNA in mRNA predictions.

RNA types	Tools	MACRO-F1^a^	MACRO-MCC	MACRO-AUROC	MACRO-AUPRC
mRNA	DM3Loc	0.4315	0.1713	0.7423	0.5743
	iLoc-mRNA	0.1832	0.0441	0.5248	0.3093
	mRNALoc	0.3441	0.0497	0.5211	0.4283
	DeepLocRNA-ind (training from scratch)	0.3191	0.0643	0.7283	0.5621
	DeepLocRNA-ind (instructive fine-tuning)	**0.4647**	**0.1774**	**0.7493**	**0.5786**
	DeepLocRNA-uni (instructive fine-tuning)	0.4075	0.158	0.7433	0.5706

a The number in bold represents the max value across different tools. The macro average represents the mean value of specific metrics across different compartments. ind: training from the independent dataset; uni: training from the unified dataset.

Subsequently, we evaluated miRNA localization using a dedicated miRNA-independent dataset. In the past, most research efforts have been directed toward developing models for mRNA and lncRNA localization prediction (Wang *et.al*. 2023). We only found iLoc-miRNA available for predicting miRNA trafficking, which offers predictions primarily distinguishing between extracellular and intracellular localization (Zhang *et al.* 2022). In our evaluation, we segregated our miRNA dataset into intracellular and extracellular segments for a comprehensive and equitable comparison with iLoc-miRNA. DeepLocRNA consistently demonstrates the beneficial contributions of pre-trained protein information, outperforming both training-from-scratch and iLoc-miRNA (Table 2). Intriguingly, all models exhibit high scores according to metrics, especially all exceeding 0.90 in AUROC (Table 2). This implies the possible existence of specific *cis-*regulatory elements within the primary sequence, facilitating the model’s adaptability to the data.

**Table 2.** Benchmarking DeepLocRNA in the prediction of miRNAs.

Tools	Cellular	Precision^a^	Recall	F1	AUROC	AUPRC
iloc-miRNA	Extracellular Intracellular	**0.8916**	0.8949	0.8932	0.9286	0.8249
		**0.9943**	0.8603	0.9225	0.9159	0.9746
DeepLocRNA (training from scratch)		0.8779	**0.9111**	0.8940	0.9438	0.8763
		0.9462	0.9117	0.9283	0.9355	0.9885
DeepLocRNA (instructive fine-tuning)		0.8850	0.9058	**0.8952**	**0.9547**	**0.8808**
		0.9531	**0.9143**	**0.9331**	**0.9419**	**0.9905**

a The number in bold represents the max value across different tools.

Finally, we evaluate our method against three other lncRNA prediction tools, including DeepLncLoc (Zeng *et al.* 2022), LncLocator (Cao *et al.* 2018), and iLoc-lncRNA (Su *et al.* 2018). While all the compared methods were trained on data with unique labels, filtering out genes with multiple labels, they displayed limited generalizability, with AUROC values ranging from 0.4904 to 0.5192 across all compartments (Table 3, Supplementary Table S6). Notably, our baseline model, trained from scratch, also outperformed these counterparts, substantiating the efficacy of our proposed model structure. Following fine-tuning of our model with pre-trained RBP interaction information, performance gains were observed across all compartments, particularly in the Exosome compartment, where the AUROC increased from 0.5690 to 0.5832. The overall performance of lncRNA localization consistently exhibits lower accuracy, despite our tool ranking as the top performer across all compartments. This suggests that reliance solely on primary sequence information may not yield robust predictions for lncRNA localization, hinting at potential limitations inherent in lncRNA trafficking. This could be influenced by unconsidered factors such as nuclear localization signal (NLS) (Hacisuleyman *et al.* 2014), nuclear retention signals (NRS) (Tripathi *et al.* 2010), or secondary structures of the sequence (Bridges *et al.* 2021).

**Table 3.** The benchmarking of DeepLocRNA in the prediction of lncRNAs.

Tools	MACRO-AUROC^a^	MACRO-AUPRC	MACRO-MCC
DeepLncLoc	0.5021	0.3349	0.0036
LncLocator	0.4965	0.3329	0
iLoc-lncRNA	0.5066	0.3355	0.0107
DeepRBPLoc (training from scratch)	0.5734	0.3586	**0.0039**
DeepRBPLoc (instructive fine-tuning)	**0.5786**	**0.3626**	**0.0039**

a The number in bold represents the max value across different tools.

### 3.2 A unified model for multi-task learning

We fine-tuned the model on the unified benchmarking dataset (Section 2), to discern crucial features from diverse RNA compositions and encapsulate the entirety of the binding mechanisms into a unified mode. This enables the training of our unified model across eight different cellular compartments spanning four RNA species (Supplementary Text).

Our unified model still slightly outperforms DM3Loc (0.7607 versus 0.7546, Supplementary Tables S1 and S2), reiterating the efficacy of our unified training approach. Furthermore, with the adoption of the unified model, we expanded the scope of prediction miRNA localization beyond the generic extracellular and intracellular categories found in iLoc-miRNA, encompassing five more specific compartments—nucleus, exosome, cytoplasm, microvesicle, and mitochondrion (Supplementary Table S2). Our model achieved AUROC scores exceeding 0.9 in the first four compartments. However, it struggled to achieve satisfactory performance in the mitochondrion compartment, likely constrained by a limited training sample size (Supplementary Table S2 and S4).

Notably, our unified model makes it possible to predict snoRNA with rare sample sizes. The AUROC values in the nucleus and cytoplasm compartments are relatively lower, standing at 0.6595 and 0.6071, respectively. Conversely, the performance in the exosome and microvesicle compartments is notably exceptional, reaching a perfect F1 score of 1 in exosome and a high value of 0.9991 in microvesicle (Supplementary Tables S2 and S4).

We also test the cross-species prediction using the mouse dataset (Section 2). It is noteworthy that our framework performs relatively well in mRNA localization and excels in generalized classification tasks, particularly in the nucleus and cytoplasm, with AUROC 0.8414 and 0.8405, respectively (Supplementary Table S5). In the context of miRNA prediction, it has strong performance in the exosome compartment, reaching AUROC 0.8125. In mRNA and miRNA prediction, our instructive fine-tuned model always performs better than the model trained from scratch in compartments with high-fidelity sample size (Table 4, Supplementary Table S5).

**Table 4.** The average performance of DeepLocRNA in mouse.

RNA species	MACRO-F1^a^	MACRO-AUROC	MACRO-AUPRC
mRNA	**0.4924 |** 0.4635	**0.7696 |** 0.7480	**0.6229 |** 0.5949
miRNA	0.9123 **|** 0.9123	**0.7140 |** 0.7020	0.5881 **| 0.5941**
lncRNA	**0.2309 |** 0.2229	0.5444 **| 0.5920**	0.4195 **| 0.4587**

a The bold numbers represent the larger values when compared with the instructive fine-tuning model (left) and training from scratch model (right).

### 3.3 Generic model explanation

**Figure 2.**
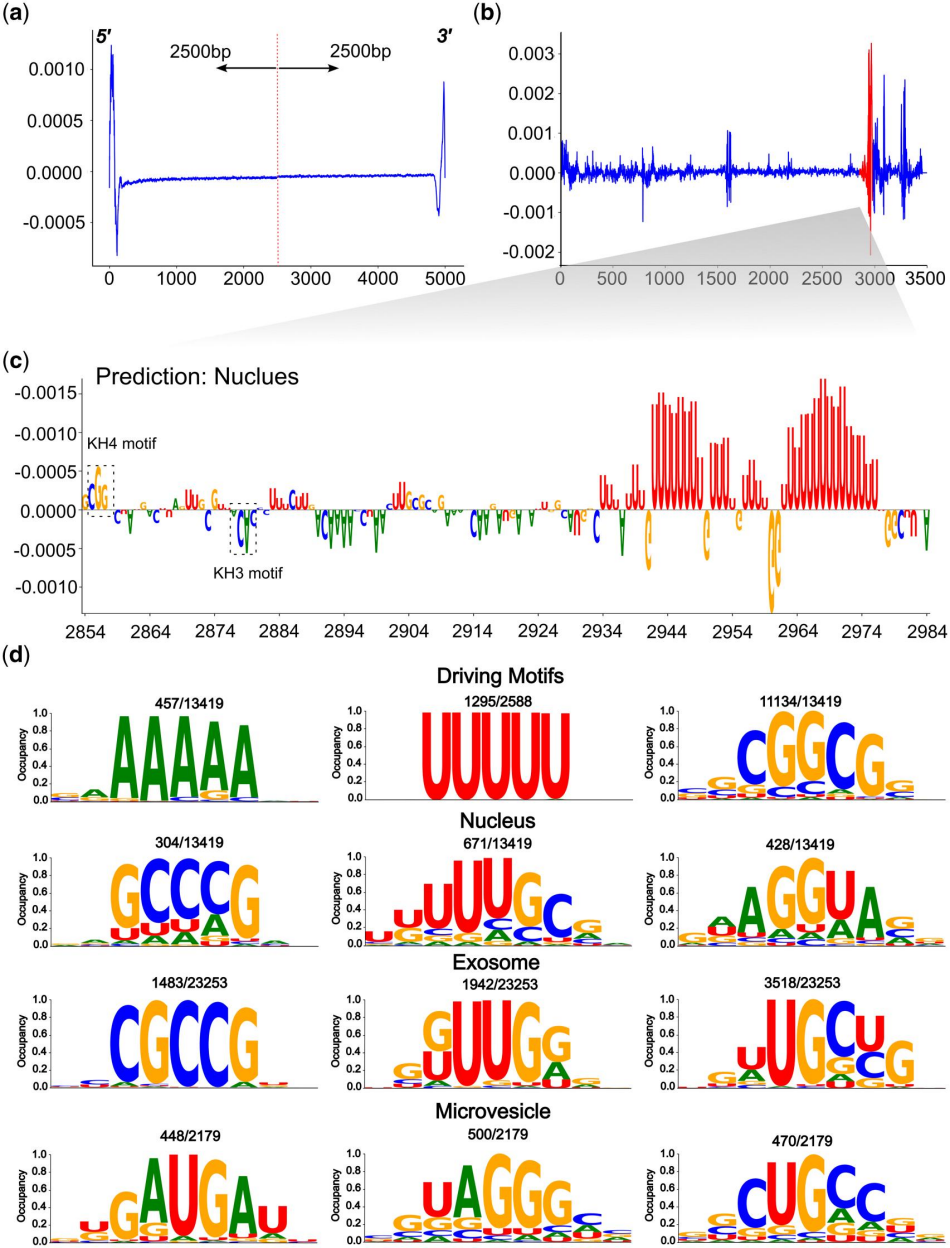
Model explanation with Integrated Gradient (IG) scores and extracted motif visualization. (a) A visual representation of the attribution score across the two ends of all the sequences in the unified dataset. Sequences exceeding 5000 nt have been truncated, resulting in a 5000 nt sequence length represented on the *x*-axis. This visualization offers insights into the attribution of importance to different regions at the sequence's beginnings and ends. (b) The IG scores for the ACTB gene. The full length of the gene sequence is displayed, with a red line indicating the zip code region within the sequence. (c) A zoomed-in version of the zipcode region from plot b. This plot showcases the attribution score across four dimensions at a single nucleotide resolution. The *x*-axis commences at the beginning of the zipcode region, allowing for a more detailed examination of the sequence's key attributes. (d) The top three 5-mer motifs within the respective localization datasets. The nucleotides displayed in the logo plot represent patterns captured as sliding windows traverse the sequences, and their attribution values are calculated using IG. The mean IG score has been normalized within a range of 0–1, as indicated on the *y*-axis. This analysis unveils crucial sequence motifs and their respective attribution values.

Integrated Gradients (IG) (Sundararajan *et al.* 2017) significantly enhances model interpretability by revealing key feature attributions linked to prediction targets, improving our understanding of the deep learning model’s decision process. Elevated scores among the four nucleotide bases signify heightened contributions of specific bases to the target compartments, culminating in the formation of a position weight matrix (PWM). We retained 2500 nt from both ends of the sequences, resulting in a total sequence length of 5000 nt for IG score calculation. Our analysis revealed consistently high attribution levels at both ends of the sequences, underscoring the substantial contributions of both the 3ʹUTR and 5ʹUTR to the localization prediction (Fig. 2a). We also attempted to calculate the attention weights of the attention layer by preserving 1000 nt from both the 5ʹ and 3ʹ ends. Our results slightly diverged from what DM3Loc found as an evenly high attention weight in two ends, which extracted and pooled features solely from the primary sequence (Wang *et al.* 2021) (Supplementary Fig. S7). In contrast, our input to the attention layer comprises abstract representations of protein-RNA interactions, suggesting a subtle shift towards a higher likelihood of RBP binding events on the 5ʹend.

### 3.4 Exploring the zipcodes in two cases

To further validate the biological significance of attribution in target prediction, we downloaded the ACTB gene from NCBI, which can be translated as the β-actin to form the actin cytoskeleton. ACTB was accurately predicted by DeepLocRNA localized as nucleus localization, even though it was not included in part of our initial benchmark dataset. Subsequently, we computed the IG scores across the full-length gene sequence and found the highest attribution was localized at 3ʹUTR of the sequence (Fig. 2b). For an in-depth examination of single nucleotide attribution, we manually curated the 52-nucleotide zipcode sequence, previously defined as the binding region for RNA-binding protein (Patel *et al.* 2012). Our investigation then focused on the model’s ability to identify motifs associated with ZBP1 and HuD, known to bind overlapping sites within the β-actin zipcode, playing a crucial role in mRNA transport (Kim *et al.* 2015). We observed a positive attribution at the beginning of the zipcode, confirming the presence of the KH4 recognition motif 5ʹ-CGGAC-3ʹ (Patel *et al.* 2012) of the RNA-binding protein ZBP1. Conversely, the KH3 recognition motif 5ʹ-ACAC-3ʹ (Patel *et al.* 2012) showed a negative attribution (Fig. 2c), implying the concurrent binding of KH3 and KH4 is improbable. Both motifs, bound by the ZBP1 protein, are located within the 52-nucleotide region. Notably, U-motifs with the highest attribution scores across the entire ACTB sequence (Fig. 2c) were found downstream of this 52-nucleotide region, likely to be bound by HuD, given their preference for U-rich features (Heinrich *et al.* 2009). When perturbing the 3ʹ ends with random nucleotides, our model does not predict the nucleus as its compartment, highlighting the robustness of our model in handling perturbation cases.

Huntington’s disease (HD) results from altered HTT gene concentration in the nucleus and cytoplasm, primarily due to expanded CAG repeats (Roos 2010). Our model can accurately predict HTT localization with variant CAG expansion levels (Supplementary Fig. S13a). Specifically, we found that increasing CAG repeats boost the prediction probabilities in both the nucleus and cytosol (Supplementary Fig. S13a), with a high level of the mutated CAG attribution (Supplementary Fig. S13c–e). Removing CAG sequences significantly reduces prediction values (Supplementary Fig. S13a). These results underline the importance of CAG in predicting HTT gene localization, which is potentially a valuable target to reduce mutant HTT mRNA accumulation and mitigate the toxic effects of the mutant protein.

### 3.5 Motif analysis

To investigate whether the modelling of RNA trafficking can unveil inherent functional elements computationally dictating localization predictions, we compute IG scores across all eight predictable compartments in our model.

The identification of the “CGGCG,” “A-motif,” and “U-motif” motifs emerges as particularly significant driven motifs, successfully classifying five out of eight compartments. Specifically, a high degree of concurrence of the “U-motif” is prominently observed in the ACTB zipcode region (Fig. 2c), indicative of a compelling binding mechanism that orchestrates the transportation of the gene from the nucleus to the cytoplasm. Furthermore, we unearth motifs specialized for specific localization. In the nucleus attribution analysis, we discerned that the expressive 5-mer motifs “GCCCG,” “AGGUA,” and “UUUGC,” which are binding motifs of RBMX (Heinrich *et al.* 2009), KHSRP (García-Mayoral *et al.* 2008), TARDBP (Volkening *et al.* 2009), regulating alternative splicing localized in the nucleus. Notably, “CGGCG” exhibits a strong correlation with the protein PPRC1, which coactivates nuclear gene transcription (Fig. 2d). “CGCCG,” found in top motifs of the exosome, is a binding motif identified by FMR1, playing a significant role in endosome cargo loading that often interacts with miRNA (Wozniak *et al.* 2020). The “GUCCG” element interacts with ZNP2, initially binding to nascent beta-actin transcripts and facilitating binding with ZBP1, associated with nuclear-to-cytoplasmic localization transport (Pan *et al.* 2007). Additionally, motifs not extensively documented in literature yet unique to certain compartments, such as “GUUUC” and “GAUGA” may potentially represent common identification patterns guiding RNA to the ER and microvesicles.

Furthermore, we conducted a comparative analysis of these compartment-specific functional motifs with the findings from RBPnet (Horlacher *et al.* 2023), which predicts the binding interactions between proteins and RNAs. Intriguingly, we identified four distinctive motifs that precisely correspond to the results previously obtained by RBPnet (Supplementary Table S3). Notably, the “U-motif” motif emerged as a prominent motif, featuring among the top two binding motifs for several proteins. For example, FUBP3 has been established as a crucial factor in the regulation of β-actin mRNA, a major constituent controlling RNA mobility and directing its localization through binding to the 3ʹ UTR (Mukherjee *et al.* 2019). As for the nuclear motif “AGGTA” NCBP2 is intricately involved in various processes, including pre-mRNA splicing, translation regulation, and nonsense-mediated mRNA decay (Gebhardt *et al.* 2015). These intriguing findings warrant further experimental exploration to elucidate the functions of these novel motifs and their interactions with relevant RBPs in the context of RNA localization.

## 4 Discussion

In this study, we address the multi-label RNA localization prediction problem by leveraging a pre-training scheme to glean protein-RNA binding characteristics at a single nucleotide resolution from the CLIP-seq data. DeepLocRNA thrives when tasked with predicting gene localization based on the guiding influence of RBPs, irrespective of RNA type. Our model also exhibits commendable generalization capabilities in cross-species prediction, particularly in distinguishing mouse mRNA between the nucleus and cytoplasm.

Furthermore, we curate a unified, nonredundant benchmark dataset encompassing four RNA types and eight distinct localizations spanning both human and mouse. To enable comparisons with other tools, we dissected the unified dataset, evaluating the performance of our method on subset data. The final model, trained on this comprehensive benchmark dataset, amalgamates sequence information in a data augmentation framework bolstered by pre-trained protein-RNA interactions. mRNA and miRNA tend to perform well, while snoRNAs show predictability despite limited data. However, lncRNAs, despite excelling in benchmarking, face challenges in achieving their full potential due to factors like alternative splicing and distinct localization patterns.

To analyze predictions, we used Integrated Gradients (IG), extracting the most informative motifs pertinent to the prediction targets through attribution methods. As a sequence-driven model, DeepLocRNA can be elucidated by examining PWMs across various RNA species, uncovering overarching patterns. These findings hold promise for experimental validation.

Our work represents a pioneering effort in creating comprehensive RNA localization prediction tools employing a sequence-driven approach, blending primary sequence information with RBP binding priors. Future enhancements may involve leveraging large RNA language models, enabling the model to grasp RNA intricacies from genome-wide nucleotide corpora and further refining RNA representation (Alipanahi *et al.* 2015). This adaptable model can also seamlessly integrate diverse data modalities, such as in-situ hybridization images, protein expression, and regulation, enhancing its robustness and applicability across various diseases and developmental contexts. Furthermore, we did not account for cell type heterogeneity in this study, primarily because of the requirement for substantial data to train our deep neural network. However, as more data becomes available in the future, it will be imperative to include considerations for cell type heterogeneity in building the model for potential applications, e.g. RNA drug delivery. The wealth of data derived from diverse sources, including microscopy images and RBP binding profiles, paves the way for the development of more precise localization prediction tools, thus facilitating drug discovery and driving novel advancements in disease treatment.

## Supplementary Material

btae065_Supplementary_Data

## Data Availability

The unified data for training and testing are available at https://zenodo.org/records/10116380. The standalone tool for local use and source code are available in the GitHub repository, https://github.com/TerminatorJ/DeepLocRNA.
