# Supplementary material for: DeepLocRNA: an interpretable deep learning model for predicting RNA subcellular localization with domain-specific transfer-learning
Source: Bioinformatics. 2024 Feb 5;40(2):btae065. doi: 10.1093/bioinformatics/btae065 (PMC10879750; doi:10.1093/bioinformatics/btae065)
Supplement: btae065_Supplementary_Data [file btae065_supplementary_data.docx]

# Supplementary Text

**Sequence Collection**

For the mRNA dataset, we simply include the mRNA benchmark dataset from DM3Loc^[1](https://www.zotero.org/google-docs/?EwTahb)^. When collecting the human and mouse datasets, we employ the following process and data source to get the sequence. For snoRNA and lncRNA, we downloaded most of the sequences from the NCBI RefSeq database ([https://ftp.ncbi.nlm.nih.gov/refseq/H_sapiens/annotation/GRCh38_latest/refseq_identifiers/GRCh38_latest_rna.fna.gz](https://ftp.ncbi.nlm.nih.gov/refseq/H_sapiens/annotation/GRCh38_latest/refseq_identifiers/G)).
Alternatively, sequences were extracted from the Ensemble database ([https://ftp.ensembl.org/pub/current_fasta/homo_sapiens/ncrna/Homo_sapiens.GR](https://ftp.ensembl.org/pub/current_fasta/homo_sapiens/ncrna/Homo_s)[Ch38.ncrna.fa.gz](file:///Users/sxr280/Documents/manuscript/2.research_articles/BIB_submission/Ch38.ncrna.fa.gz)), and RNAcentral ([https://ftp.ebi.ac.uk/pub/databases/RNAcentral/current_release/sequences/rnacentral_species_specific_ids.fasta.gz](https://ftp.ebi.ac.uk/pub/da)) if they were not present in NCBI. The miRNA sequence was downloaded from the miRBase (<https://www.mirbase.org/>). To remove the confusion of the gene names and their synonyms in the dataset, we downloaded the FTP file ([https://ftp.ncbi.nlm.nih.gov/refseq/H_sapiens/Homo_sapiens.gene_info.gz](https://ftp.ncbi.nlm.nih.gov/refseq/H_sapiens/Ho)) to match the unique NCBI id and gene symbol for each gene when available in the NCBI. Alternative splicing selectively includes or excludes exons while RNA processing, resulting in variant transcripts of a certain gene. If the transcript IDs were not provided, we selected the longest one.

## **Unified benchmarking dataset**

After getting all sequences that align completely with the localisation label provided by RNALocate 2.0, we built a comprehensive dataset for humans and mice, including mRNA, lncRNA, miRNA, and snoRNA. To specify the target, we selected and eliminated redundant representations, assigning the labels “Nucleus”， “Chromatin”， “Nucleoplasm”, “Nucleolus”, and “Nuclear” with the same target label as "Nucleus". Like DM3Loc^[1](https://www.zotero.org/google-docs/?azmj5K)^, we also kept cytoplasm to assist training by data augmentation, while clearing it in the testing step. The single-label genes were mixed with multilabel genes to form a rich data source for learning the representative localisation patterns. Consequently, the pooled 8 most abundant target compartments were kept. To prevent data leakage, we employed CD-HIT-EST^[2](https://www.zotero.org/google-docs/?9VPHEe)^ to eliminate redundant sequences. We employed a two-step process to eliminate redundant sequences when pooling all sequences. In the first step, we filtered the intra-dataset by establishing selection criteria, which included a similarity threshold of 0.9 for lncRNA and 0.95 for miRNA, and snoRNA. These criteria considered factors such as abundance and sequence length. Sequences of mRNA were preprocessed to be non-redundant, as described in a prior work^[1](https://www.zotero.org/google-docs/?xiuYQx)^, with a cut-off of 80% sequence similarity. In the second step, we amalgamated all the sequences and set a similarity threshold of 0.95 to retain most of the short sequences. This two-step approach is designed to preserve most short sequences ($<$ 100nt), as it filters out the most similar long sequences in the first step while allowing individual short sequences to form distinct clusters in the second step.

This process resulted in the following number of remaining genes within a specific compartment: Nucleus (13,352), Exosome (22,335), Cytosol (2,587), Cytoplasm (10,026), Ribosome (5,226), Membrane (3,356), ER (1,977), Microvesicle (1,958), and Mitochondrion (33) (Supplementary Table 4), and the mRNA takes the majority part of the final filtered genes in each compartment (Supplementary Figure 5). This comprehensive pooled benchmarking dataset was used to train a unified model in which four RNA species were involved across 8 compartments. Curated datasets were split into 5-fold subsets according to the RNA types and the distribution of the constitution of localisation. For example, genes with labels as “111000000”, which means they have the label of Nucleus, Exosome, and Cytosol, will be split accordingly in mRNA and miRNA if they exist in these two RNA species. Otherwise, only one of them will take each fold.

## **Independent benchmarking dataset**

To assess the performance of various methods applied to a specific RNA type, we curate a subset of RNA data from our unified benchmark dataset for rigorous benchmarking analysis. This benchmark dataset exclusively comprises RNA localisation data sourced from Homo sapiens, encompassing 7 distinct cellular compartments in mRNA, namely the Nucleus, Exosome, Cytosol, Cytoplasm, Ribosome, Membrane, and ER. In the case of lncRNA, we focus on 5 compartments: Nucleus, Exosome, Cytosol, Cytoplasm, and Membrane. miRNA was labelled as extracellular and intracellular to match the comparison with iloc-miRNA^[3](https://www.zotero.org/google-docs/?QHiSvQ)^. snoRNA are not subjected to division due to the absence of predictive tools for comparison. Cytoplasm was kept when cytosol didn’t exist in the testing scenario.

Our independent dataset primarily comprises data from three RNA types: mRNA, lncRNA, and miRNA. mRNA data exhibit 58 distinct label combinations, ranging from single labels to a maximum of seven labels. The Exosome label stands out as the most popular label and is nearly twice as prevalent as the next most popular label, which is a combination of both nucleus and cytoplasm. Similarly, in lncRNA, we observe 21 label combinations, yet none of the lncRNAs are associated with the ER and membrane compartments. However, we also found 21 combinations in miRNA which are also dominated by a combination of exosome and microvesicle (Supplementary Figure 3).

**Mouse dataset**

Mouse sequence data were processed the same as it was implemented in the human unified dataset, including reducing the redundant sequences and training test split. After carefully selecting the curated mouse data, we get the integrated dataset with three RNA types, lncRNA, miRNA, and mRNA, across 4 variant compartments, Nucleus 2271, Exosome 1116, Cytoplasm 1520, Mitochondrion 96 (Supplementary Table 4). Here we kept the cytoplasm because the cytosol compartment only has dozens of genes that are filtered out in our selection stage. All data were split into 5-fold and used for cross-validation. No tools are available for predicting mouse RNA localisation, so we didn’t split the mouse unified benchmarking dataset into subsets to compare different methods.

### **RBP backbone model**

Transcript localisation is largely governed by an intricate interplay of RNA-binding proteins (RBPs)^[4](https://www.zotero.org/google-docs/?okVMnH)^. Consequently, RBP-binding information is expected to boost the accuracy of localisation predictions. To obtain the RBP binding signal, derived from the interaction between RNAs and RBPs that principally guides the RNA trafficking to the target compartments, a pre-trained RBP model was built. We selected 8000 nt as our input length. For sequences longer than 8000 nt, we truncated both ends to keep both 5’ and 3’ information. Sequences shorter than 8000 nt were padded to reach this length. As a result, all sequences were standardized to 8000 nt, ensuring a consistent input length. We take an 8000 nt RNA sequence as input, which is one-hot encoded by mapping the bases A, C, G and U to binary vectors [1, 0, 0, 0], [0, 1, 0, 0], [0, 0, 1, 0] and [0, 0, 0, 1], respectively. The architecture of our RBP backbone model was adapted from RBPNet^[5](https://www.zotero.org/google-docs/?rh90Wc)^, and the architecture is as follows. First, an initial 1D convolution layer with 512 filters of size 7 act as a motif extract and project the RNA sequence into a high-dimensional space. This is followed by 10 residual blocks, each containing a 1D convolution operation with filters of size 4, batch normalization, ReLU activation and dropout with a probability of 0.3. The first 6 residual blocks contain 384 filters, while the last 4 blocks contain 512 filters. Later, the output of the last residual block then serves as input to 256 output heads (Supplementary Figure 6). A dilation factor of 1.5^i is applied to the convolution operation of each block, where i = {0, …, 9} is the depth of the residual block. Finally, a point-wise convolution operation with 223 filters and linear activation projects the feature map of the last residual block to logits parametrizing a multinomial distribution of eCLIP crosslink counts, in analogy to RBPNet^[5](https://www.zotero.org/google-docs/?5Bm7T2)^. The loss was computed as the sum of negative log-likelihoods across the 223 eCLIP tracks. For the training set construction, the human transcriptome CRCh38.p13 was first tiled into fixed-length 1000 nt regions using a sliding-window approach with a stride of 750. For each region, the corresponding RNA sequence together with count-vectors for each eCLIP track were extracted. Samples were then split chromosome-wise into train, validation and test sets as RBPNet^[5](https://www.zotero.org/google-docs/?UFWan1)^.The model was trained for 50 epochs using the Adam optimizer with a learning rate of 0.001. When doing fine-tuning, a pooling layer with a size of 8 was introduced, leading to an effective sequence length of 1000.

## **Model construction**

The workflow of our model construction begins with a backbone model that is pre-trained to predict RBP-binding profiles for a set of eCLIP datasets from the ENCODE database^[6](https://www.zotero.org/google-docs/?KKEqhr)^, followed by an attention layer and a fully connected layer, before the classification heads. In the initial pre-training stage, the model is encouraged to detect RBP binding sites, thereby learning a rich representation of an RNA sequence, conditioned on its trans-acting factors. We hypothesize that the pre-trained hidden representations encapsulate the broadly underlying principles governing RBP-RNA interactions, thereby learning a rich representation of an RNA sequence conditioned on its trans-acting factors. Potentially, a subset of these interactions plays a crucial role in guiding RNA localisation.

To achieve this objective, we undertook a series of technical steps to modify the backbone model. Firstly, we removed the output heads from the backbone model to extract critical RBP binding information. As a result, the model generated embedding vectors of the shape [8000,256] (Supplementary Figure 6). Subsequently, an attention layer was strategically incorporated to capture the salient regions within the sequences. Additionally, a fully connected layer was added to further extract the feature, along with new classification heads to facilitate RNA localisation prediction.

DeepLocRNA was subsequently fine-tuned with a rich diversity of RNA localisation data, encompassing lncRNA, miRNA, snoRNA, and mRNA for multi-RNA localisation prediction (Supplementary Table 4). During the fine-tuning of the model, we observed optimal performance when half of the intermediate layers were unfrozen, allowing the model to learn localized and contextually relevant RBP binding representations (Supplementary Figure 1).

#

### **Attention block**

To enhance the model's focus on the functional regions of the sequence, DeepLocRNA incorporates an attention mechanism, a widely utilized technique in various fields including document classification^7^. The attention mechanism is comprised of three fundamental components:

The score function calculates attention scores and is defined as follows:

| $e=M(W_{2}tanh(W_{1}H))$ | $(1)$ |
| --- | --- |

In this scenario, we suppose the attention score function takes the output of the backbone model as the input matrix $H$ $\in$ $R^{256\times T}$, where 256 is the final embedding dimension of the backbone model and $T$ is the sequence length after pooling (1000 nt). To get the attention score $e$, the score function was introduced, where $W_{1}$ $\in$ $R^{a\times256}$ serves as a weight matrix and a is the attention hidden dimension hyperparameter. $W_{2}$ ∈ $R^{h\times a}$ defines the head of the attention mechanism, where $h$ represents the attention heads. To introduce non-linearity and facilitate gradient propagation, the *Tanh* function is employed. After the multiplication of matrixes, we can get the score value $e$ $\in$ $R^{h\times T}$. Furthermore, the mask vector $M$ plays a crucial role by imposing a substantial penalty of -10000 on the padded sequence, effectively diverting the attention away from these regions. Specifically, we used $L1$-regularization to $W_{1}$ and $W_{2}$ to prevent overfitting with the weight as 0.001.

Next, the attention scores are further processed to be probability within [0, 1] by the sigmoid function before smoothing across the sequence length $T$ to get the attention weight.

| $A= \frac{sigmoid(e_{i,j})}{\sum_{j=1}^{T} sigmoid(e_{i,j})}*\alpha$ | $(2)$ |
| --- | --- |

Where $\alpha$ is the proportion of effective sequence to the pooled fixed length $T$ (1000 nt). The normalized attention weights are then multiplied by the input matrix to get the context value $M$ $\in$ $R^{256\times h}$.

| $M = {HA}^{T}$ | $(3)$ |
| --- | --- |

**Gradient clip**

To stabilize the training process and prevent gradient-related challenges, we applied a gradient clip during backpropagation. Specifically, the gradient was stored as a vector and the L2 norm of the gradient was calculated.

We then check whether the L2 norm of the gradients exceeds a predefined threshold (chosen to be 1). If the L2 norm of the gradient ${\parallel\nabla L(\theta) \parallel}_{2}$ exceeds the thresholds, we rescale the gradient as follows:

| ${\parallel\nabla L(\theta) \parallel}_{2} <threshold$ | (4) |
| --- | --- |

| $\nabla L_{clipped}(\theta)=\frac{threshold}{{\parallel\nabla L\left( \theta\right)\parallel}_{2}}* \nabla L(\theta)$ | (5) |
| --- | --- |

This step ensures that the magnitude of the gradients is under control, preventing any exploding gradients while training.

#

# **Benchmarking the models**

During the benchmarking of mRNA predictive tools, there was a potential overlap between the training data used for iLoc_mRNA, mRNALoc and our benchmark test dataset. The data leakage could artificially improve model performance and cause the benchmark analysis to become unfair. Therefore, we excluded the genes in our benchmark test that were involved in iLoc_mRNA and mRNALoc training step while doing benchmark comparison.

Specifically, to mitigate this issue, we filtered out approximately 900 genes from each fold of the test data used for iLoc_mRNA. Additionally, when calculating performance metrics, we excluded multiple label predictions generated by iLoc_mRNA and instead focused on four unique labels - Nucleus, Cytosol, Ribosome, and ER - for binary evaluation while setting the cutoff as 0.5. To evaluate mRNALoc, we accessed the webserver and downloaded the standalone tool from <http://proteininformatics.org/mkumar/mrnaloc/download.html>. We removed any benchmark test dataset overlapping with their training data. Any predictions labelled as “Extracellular_region”, “Mitochondria”, and “No Location Found” were assigned a value of “0” to differentiate them from the true label. Predictions within our benchmark dataset labels were assigned their predicted values. We set a cutoff of 0.1 for mRNALoc when evaluating using the benchmark test dataset. The standalone version of DM3Loc was downloaded from <https://github.com/duolinwang/DM3Loc>. To parallel evaluate DM3Loc, we retrained the model in 5-fold cross-validation with the same non-redundant multilabel benchmark dataset.

In the evaluation of lncRNA predictive performance, we employed a suite of tools, including LncLocator (<http://www.csbio.sjtu.edu.cn/bioinf/lncLocator/>), DeepLncLoc ([https://github.com/](https://github.com/CSUBioGroup/)[CSUBioGroup/](file:///Users/sxr280/Documents/manuscript/2.research_articles/BIB_submission/CSUBioGroup)DeepLncLoc), and iLoc-lncRNA (<http://lin-group.cn/server/iLoc-LncRNA/>predictor.php). In cases where downloading the standalone tools proved unfeasible, we resorted to utilizing their respective web servers to conduct the predictions.

For the benchmarking of miRNA, we relied on the web server predictions of iloc-miRNA. To facilitate the evaluation process, we categorized all compartments into two overarching groups: extracellular and intracellular, and computed the relevant metrics separately for these distinct partitions.

#

# Supplementary Figures


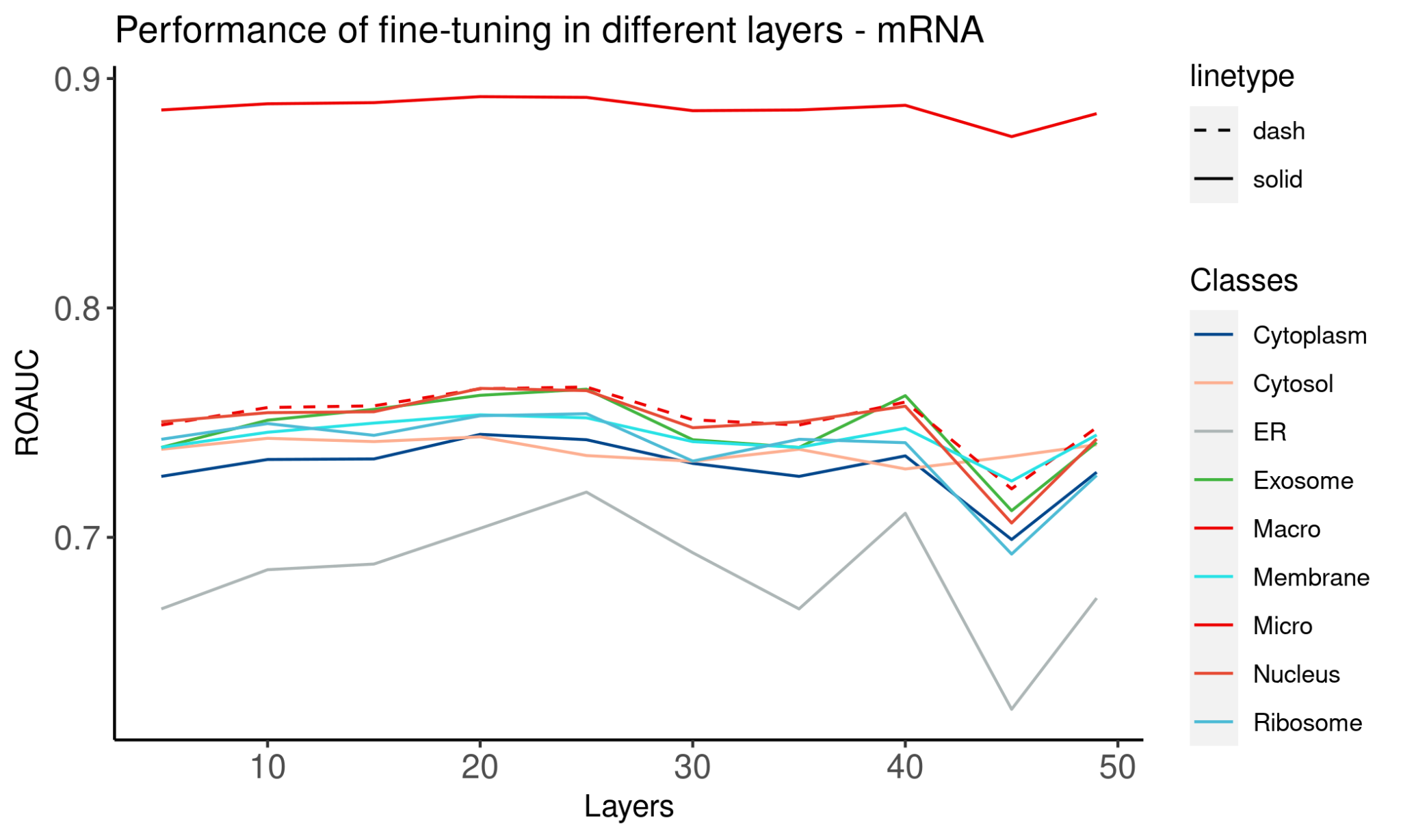


**Supplementary Figure 1**. The results of fine-tuning the backbone model. We assess the performance of the DeepLocRNA model in downstream localisation prediction by releasing different layers of the backbone model. The x-axis depicts the layers that release from the end of the backbone model to the stem layer, where smaller values indicate higher retention of RBP-RNA binding information. Each compartment is represented by a different colour, except for the two average metrics - macro average and micro average - shown in red. The micro average is visualized as a dashed line, while the macro average is presented as a solid line for distinction.


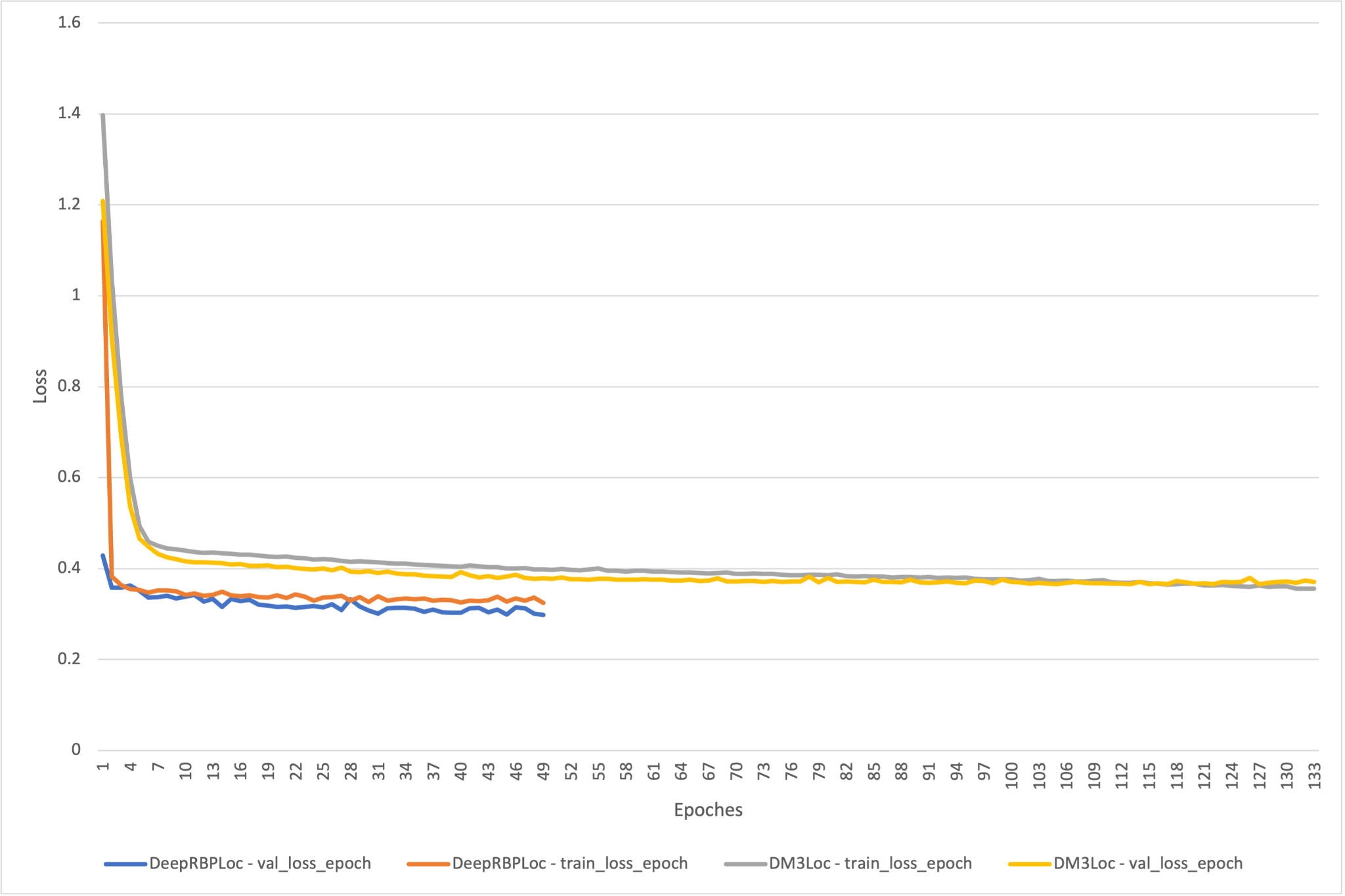


**Supplementary Figure 2.** The loss curve during the training of DeepLocRNA and DM3Loc. the x-axis represents the number of epochs needed for model convergence. the y-axis shows the decrease of the binary cross entropy loss while training.


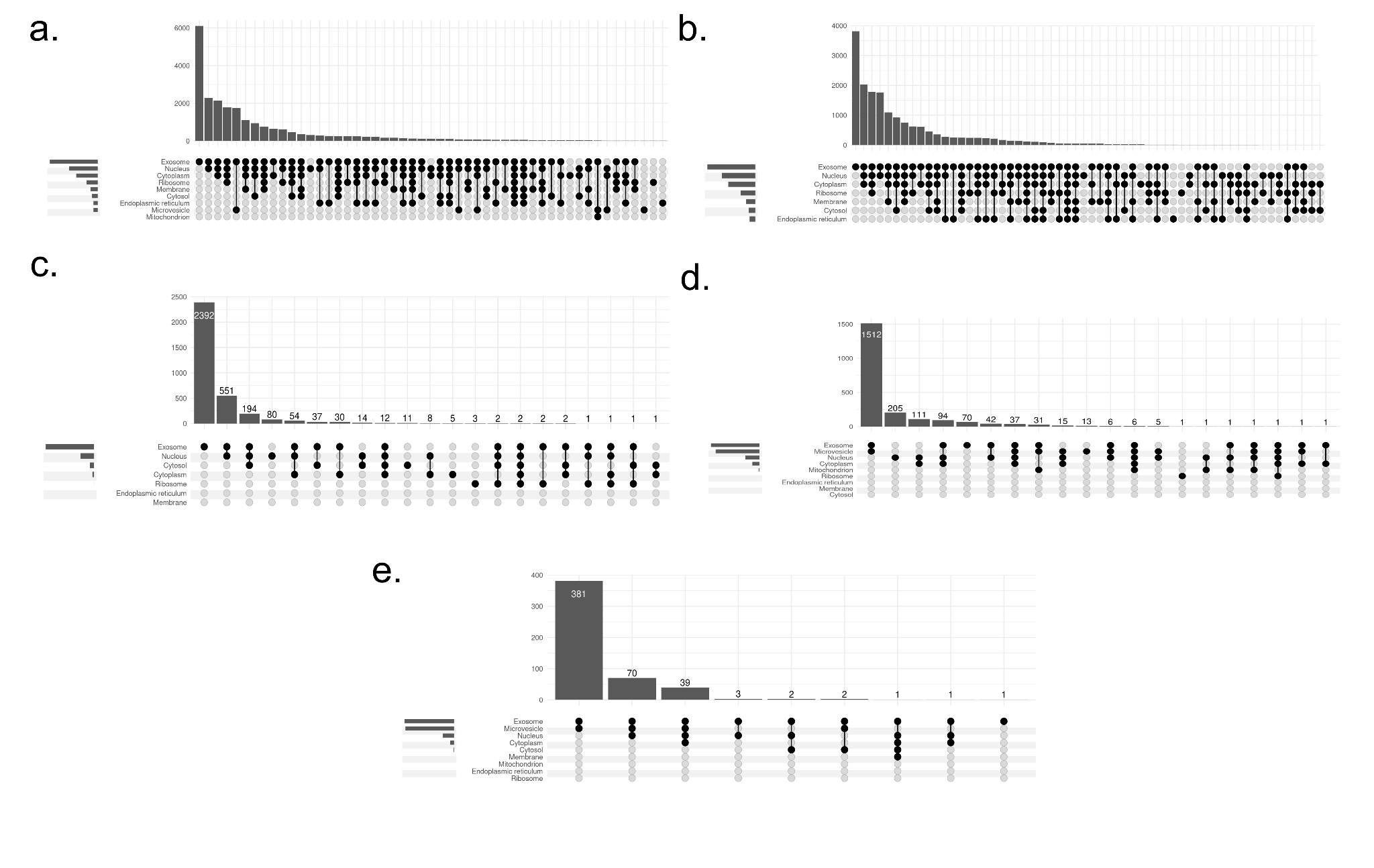


**Supplementary Figure 3.** Visualization of the multilabel data. These UpSet plots show different combinations of RNA localisations in our dataset. Each column represents a case of multiple labels. The bar in each column indicates its abundance and the tiny legend on the left side of each plot represents the abundance of each compartment. **a**) all RNA; **b**) mRNA; **c**) lncRNA; **d**) miRNA; **e**) snoRNA.


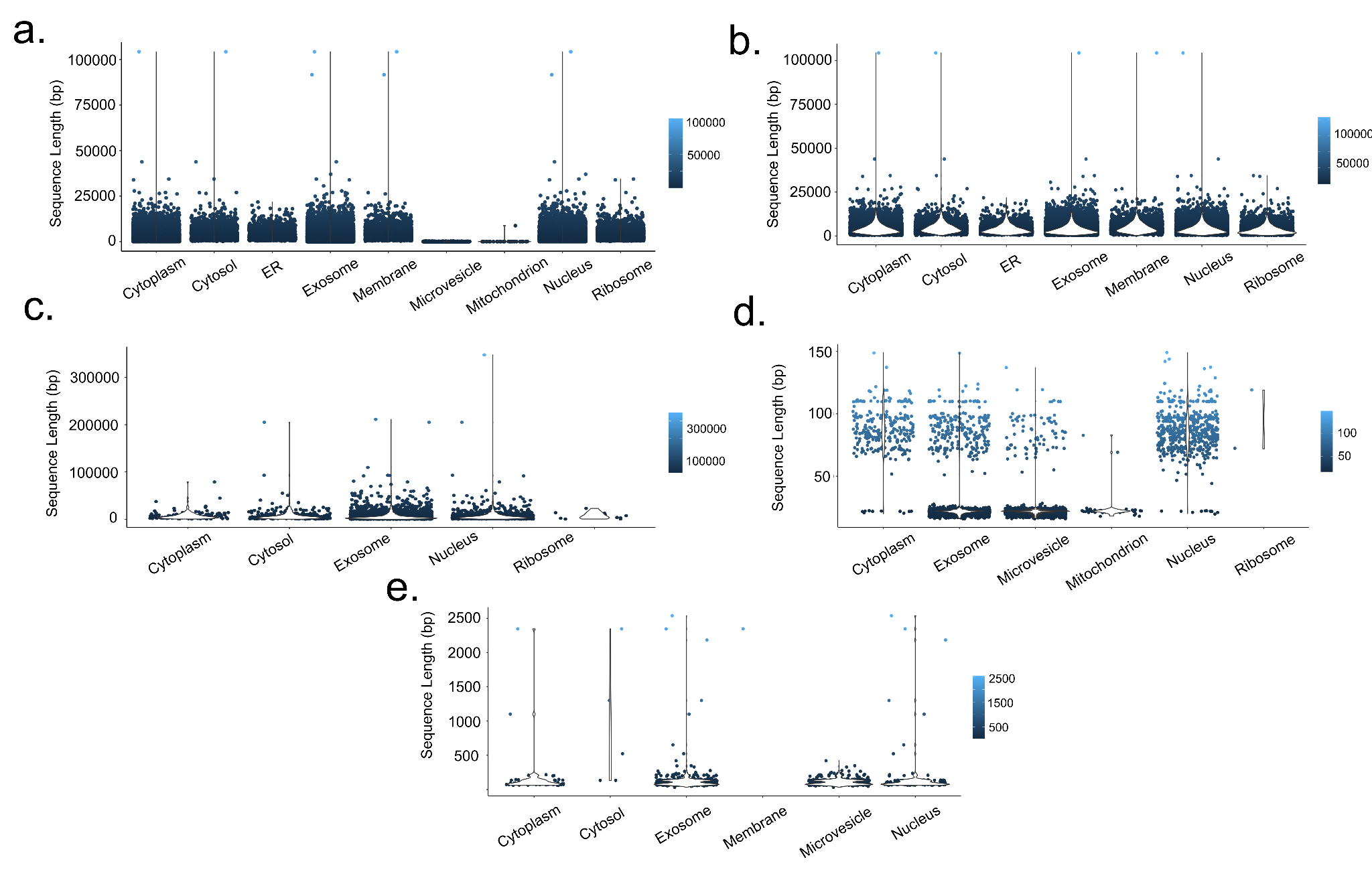


**Supplementary Figure 4.** Violin plots of sequence length distribution across all available compartments. **a**) all RNA; **b**) mRNA; **c**) lncRNA; **d**) miRNA; **e**) snoRNA.


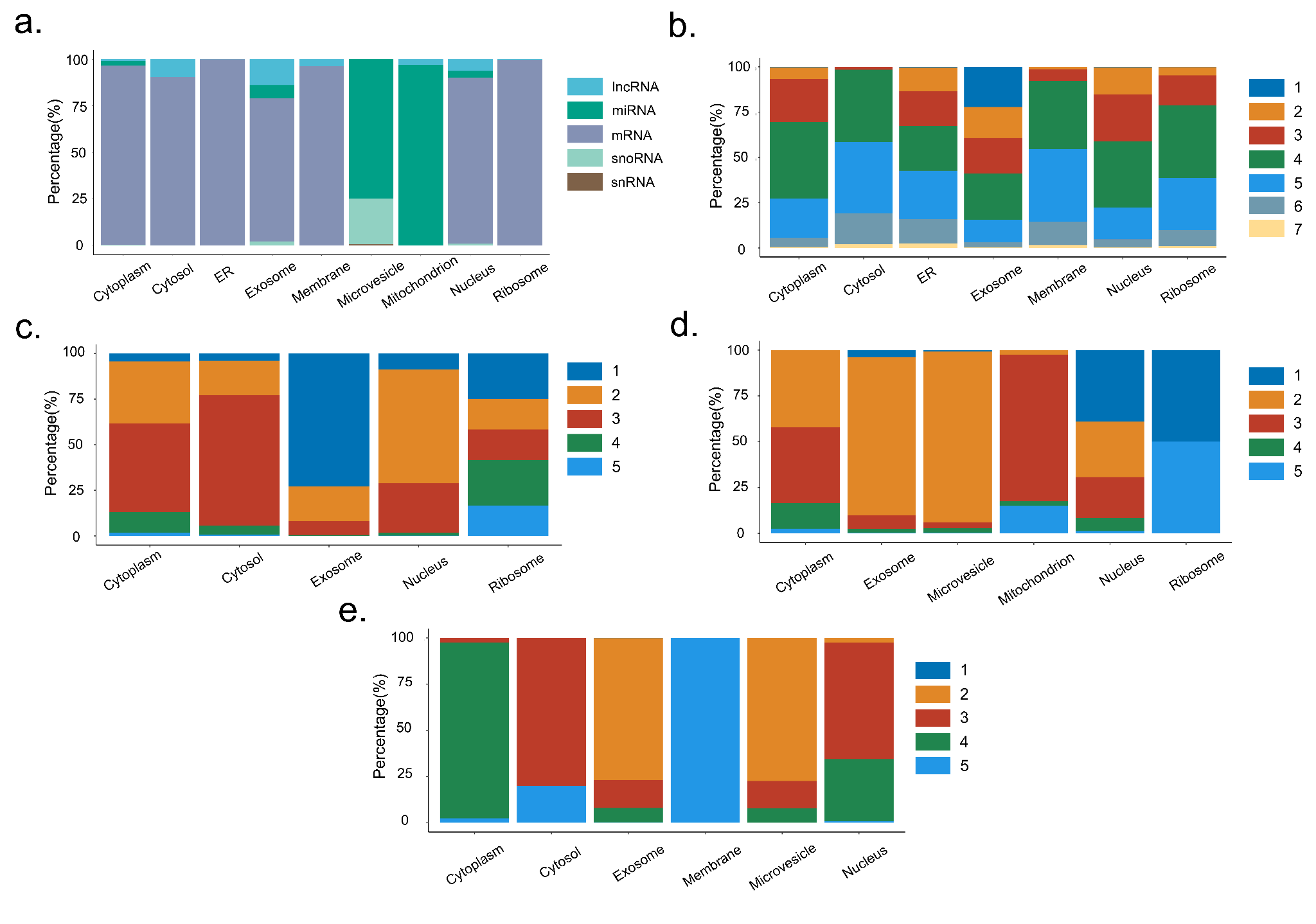


**Supplementary Figure 5.** The dispersion degree of each compartment. **a**) Abundance of RNA distributed across each compartment. **b**, **c**, **d**, **e**) The number of compartments with which a single compartment can interact in multilabel target data. The plots represent the following RNA types: mRNA, lncRNA, miRNA, and snoRNA, respectively.


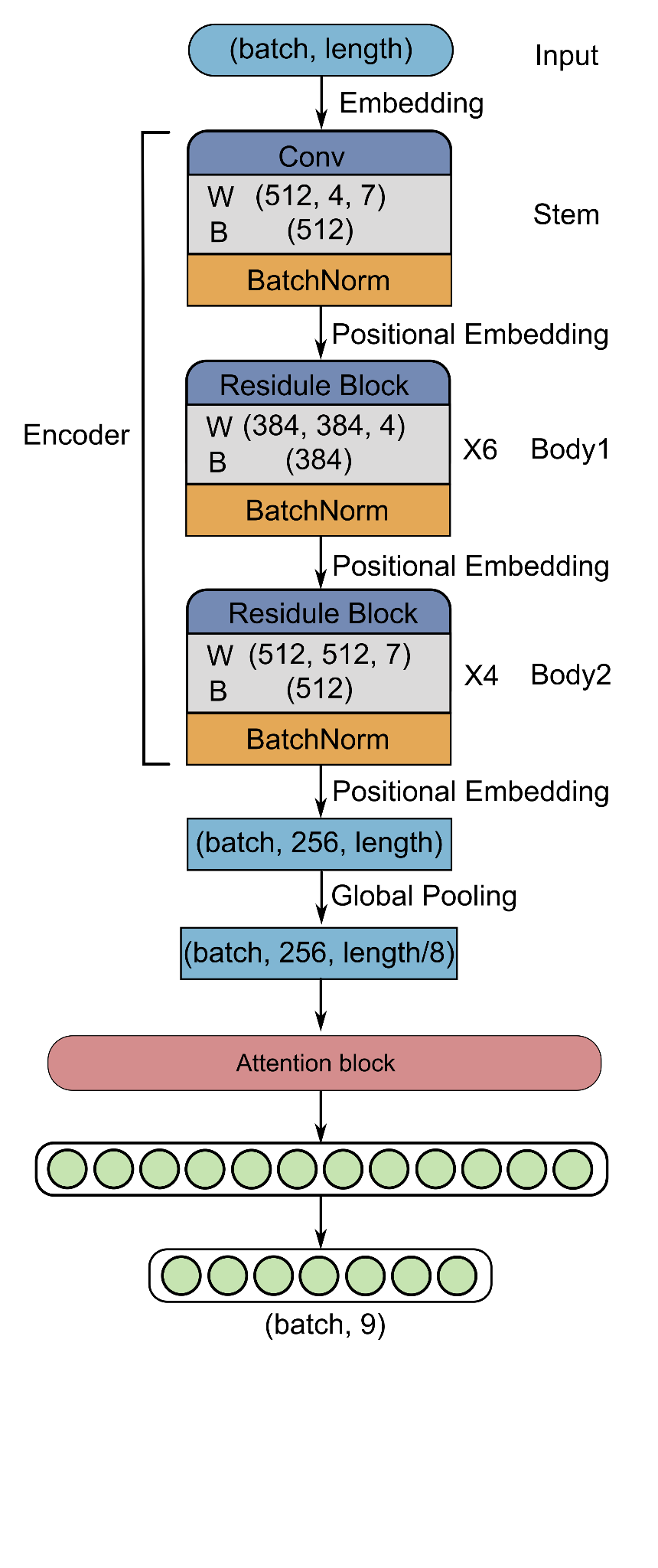


**Supplementary Figure 6.** The model architecture of DeepLocRNA**.**


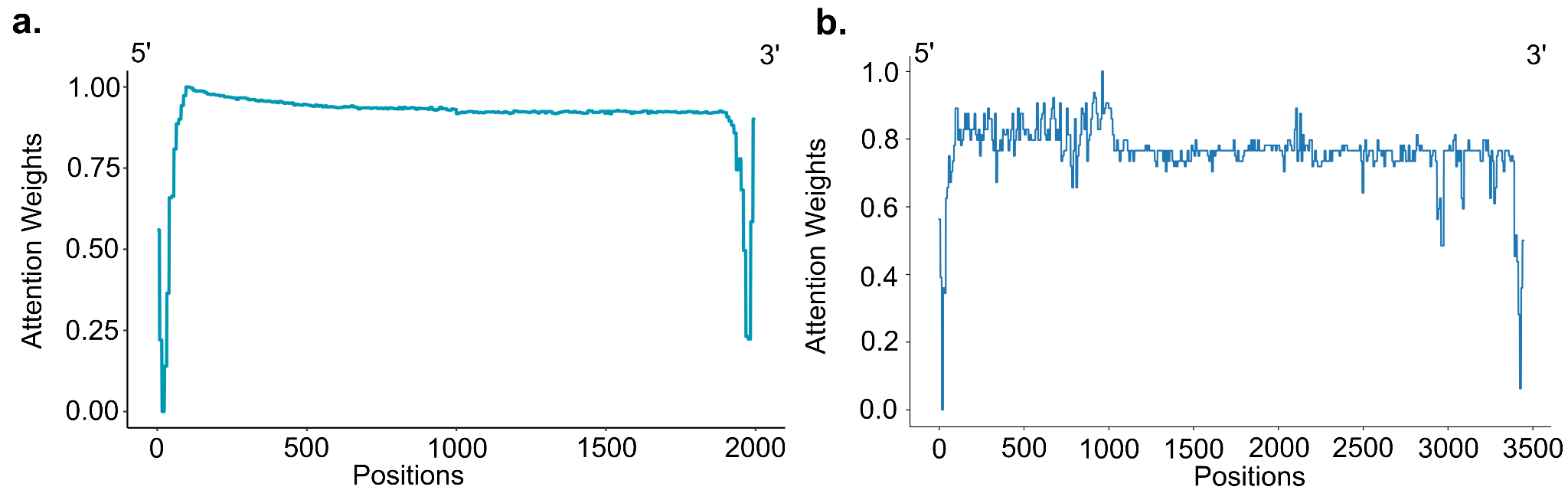


**Supplementary Figure 7.**  Attention weights analysis across the primary sequences. **a**) This plot displays the attention weights extracted from the attention layer, focusing on the two ends of the sequence. These attention weights provide insights into the neural network's concentration on specific elements at the beginning and end of the sequence, shedding light on the model's processing priorities. **b**) The attention weights for the ACTB gene are visualized across its entire sequence. This comprehensive view allows for a detailed examination of how the model allocates attention to different segments of the gene.

**
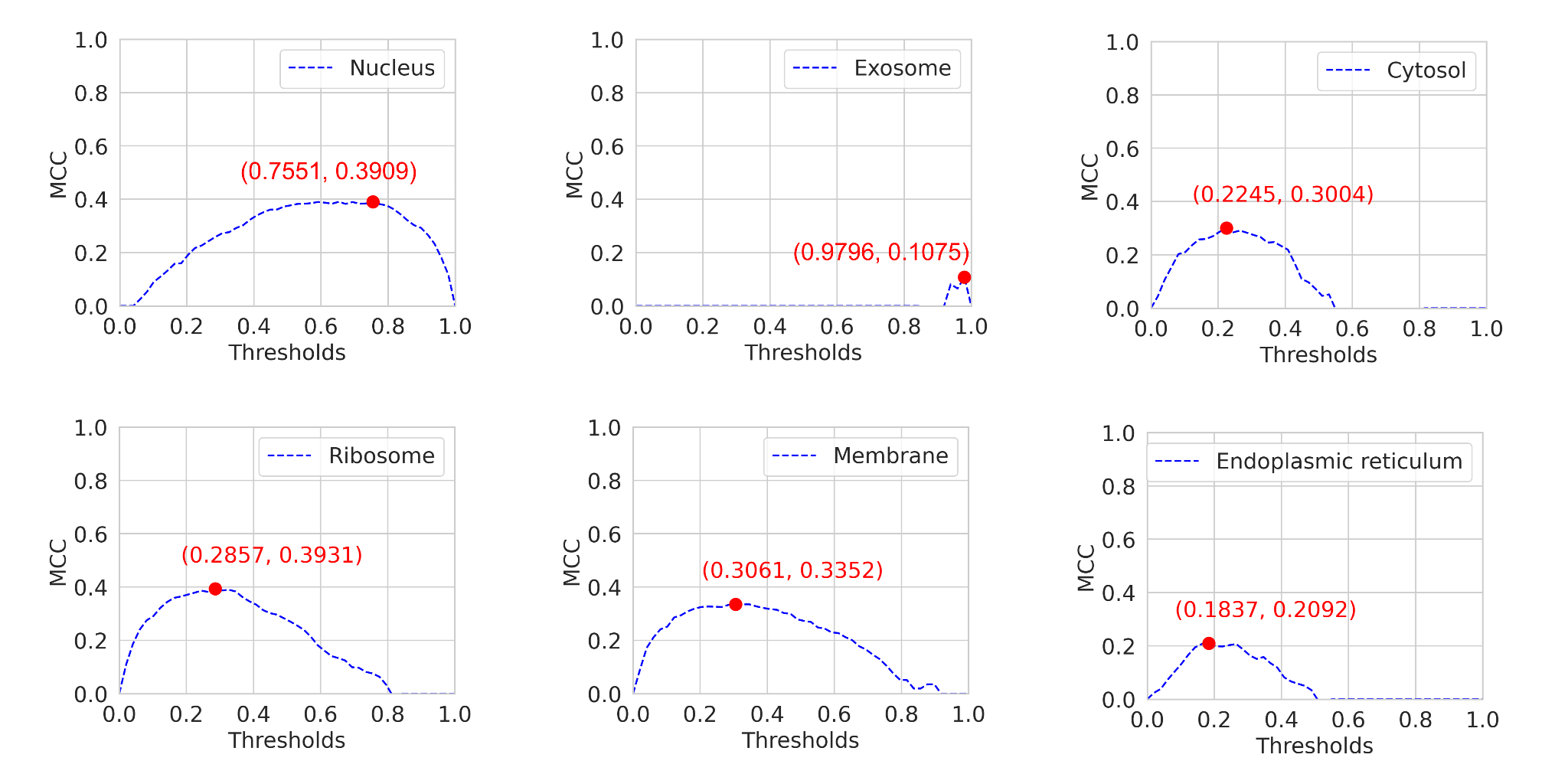
**

**Supplementary Figure 8.** The best MCC score of mRNAs across 6 different compartments. The thresholds were initialized by evenly dividing 50 numbers from 0 to 1. The coordinates in red represent the best thresholds and MCC values.

**
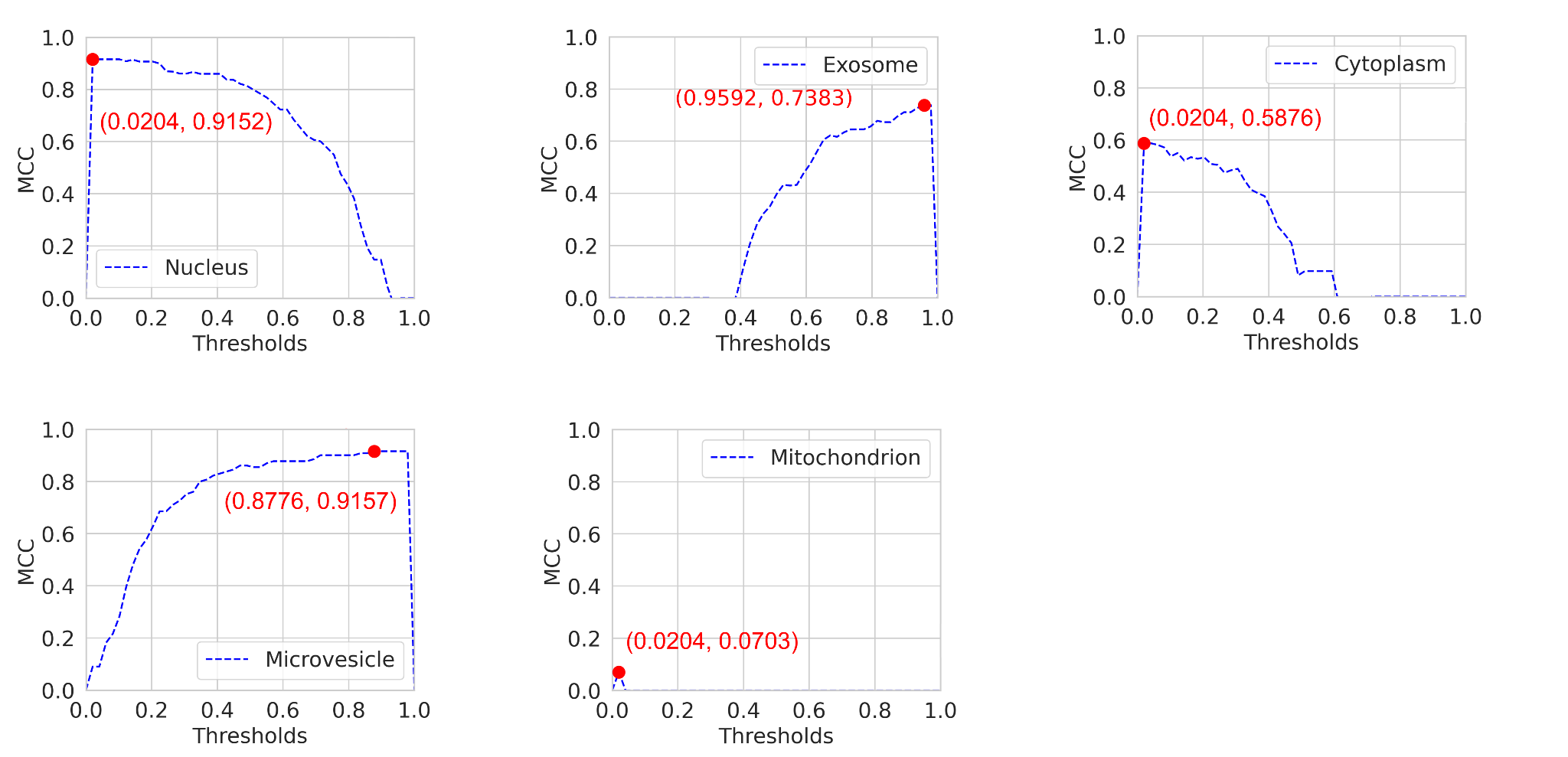
**

**Supplementary Figure 9.** The best MCC score of miRNAs across 5 different compartments. The thresholds were initialized by evenly dividing 50 numbers from 0 to 1. The coordinates in red represent the best thresholds and MCC values. Compartments with genes less than 20 were removed. Cytoplasm was shown to replace cytosol because of its rare data.


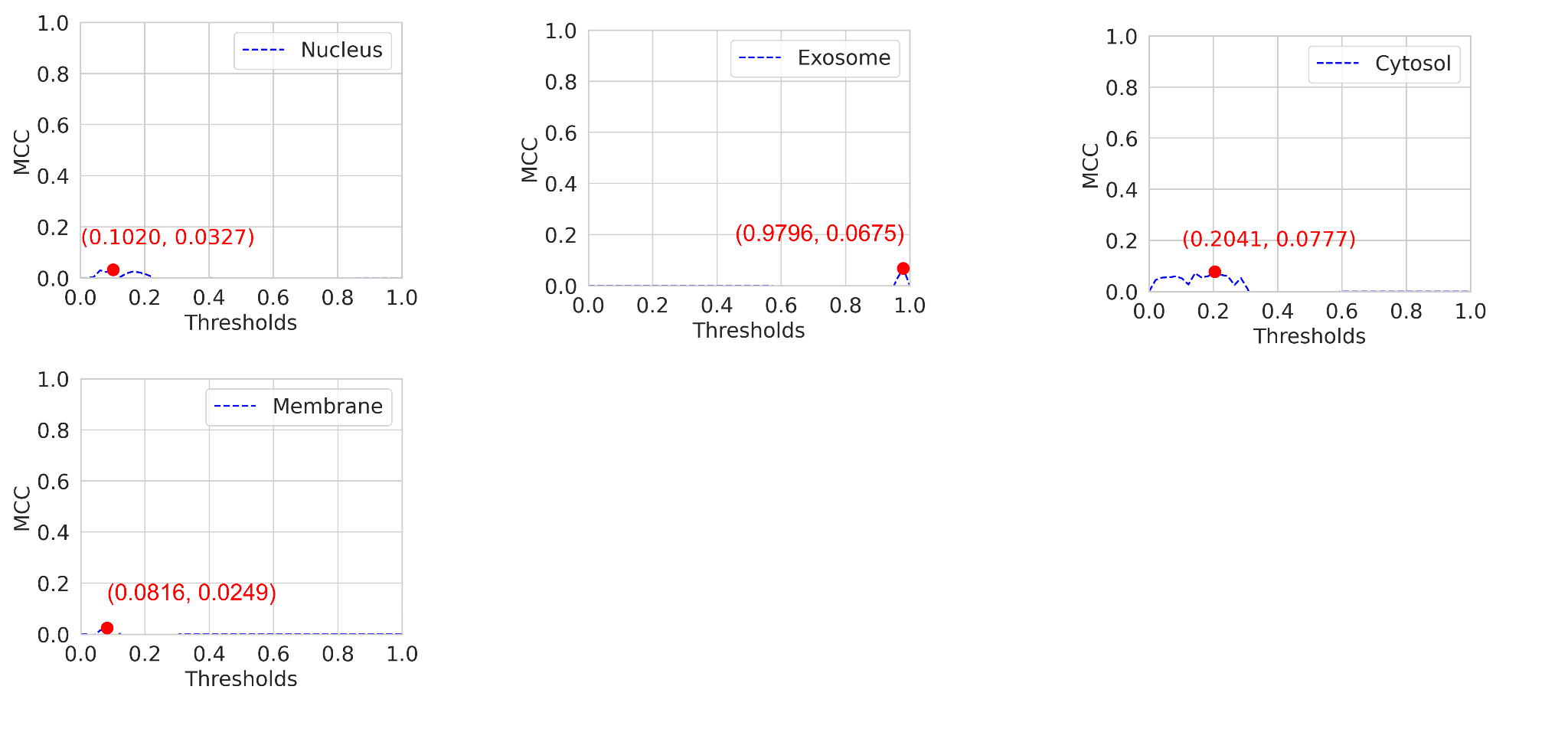


**Supplementary Figure 10.** The best MCC score of lncRNA across 4 different compartments. The thresholds were initialized by evenly dividing 50 numbers from 0 to 1. The coordinates in red represent the best thresholds and MCC values. Compartments with genes less than 20 were removed.


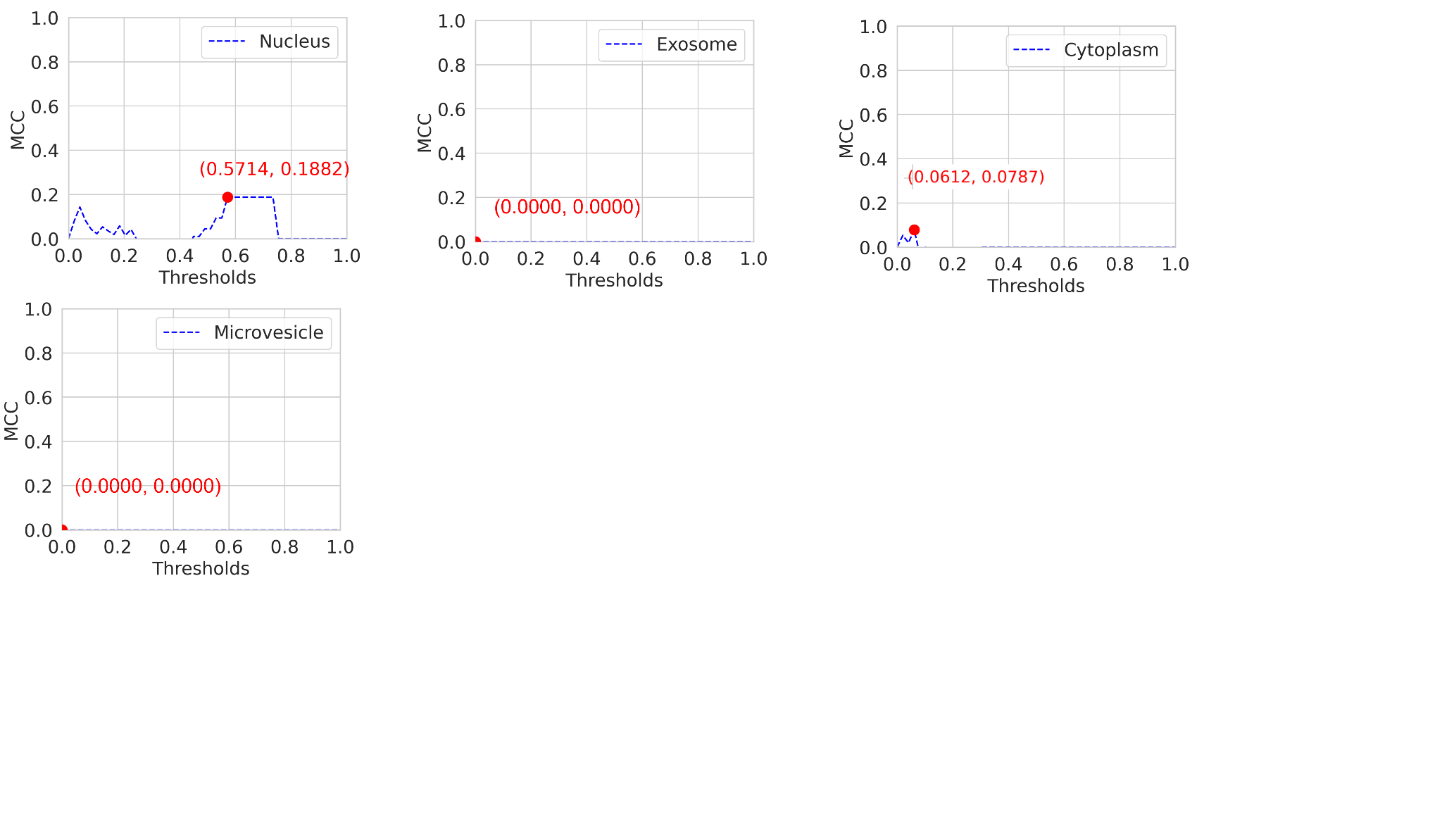


**Supplementary Figure 11.** The best MCC score of snoRNA across 4 different compartments. The thresholds were initialized by evenly dividing 50 numbers from 0 to 1. The coordinates in red represent the best thresholds and MCC values. Compartments with genes less than 20 were removed, Cytoplasm was shown to replace cytosol because of its rare data.


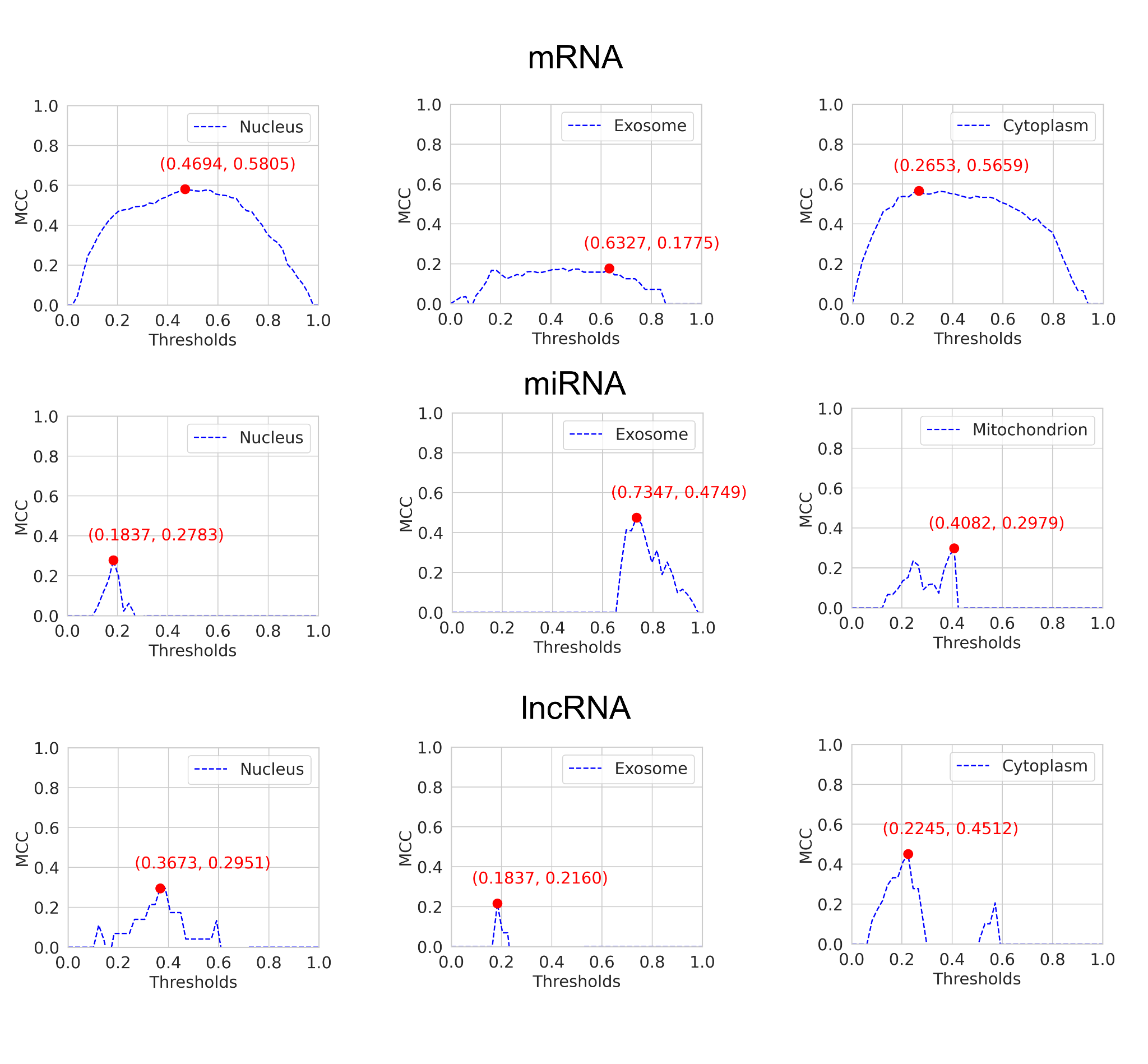


**Supplementary Figure 12**. The best MCC score of 3 RNA types in the mouse model. The thresholds were initialized by evenly dividing 50 numbers from 0 to 1. The coordinates in red represent the best thresholds and MCC values. Compartments with genes less than 20 were removed, Cytoplasm was shown to replace cytosol when it has rare data.


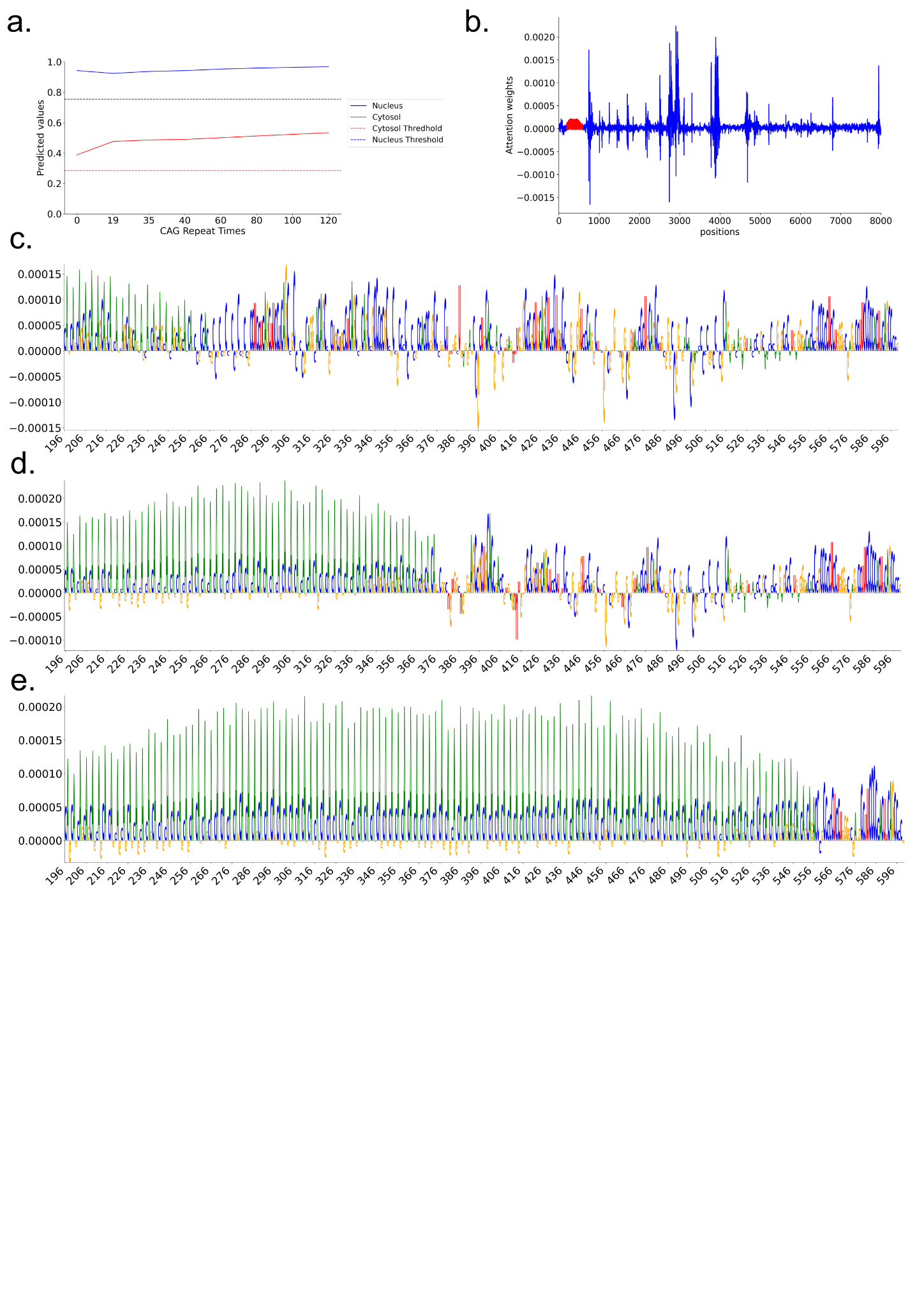


**Supplementary Figure 13.** Analysis of the attribution of expanded CAG repeats. **a**) Variant times of CAG repeats and corresponding prediction probabilities by DeepLocRNA. On the x-axis, '19' represents the initial CAG repeat at the 5’ end of the HTT gene, while '0' signifies the complete replacement of all CAG repeats in the HTT gene with random nucleotides. **b**) IG values along the entire HTT gene sequence, with red regions denoting perturbed CAG repeats. **c**, **d**, **e**) Presentation of three levels of expanded CAG repeats and their attribution scores post-mutation, with '19,' '60,' and '120' repeats of CAG shown, respectively.

| **Tools** | **Compartments** | **F1** | **MCC** | **AUROC** | **AUPRC** |
| --- | --- | --- | --- | --- | --- |
| DM3Loc | Nucleus  Exosome  Cytosol  Ribosome  Membrane  ER | 0.8256  0.9959  0.0242  **0.4680**  0.264  0.011 | 0.3516  0  0.0625  **0.3096**  0.2589  0.045 | 0.7745  0.7273  0.7399  **0.7592**  0.7546  0.6980 | 0.8763  0.9964  0.3203  **0.5528**  **0.4439**  0.2563 |
| iLoc-mRNA | Nucleus  Cytosol  Ribosome  ER | 0  0.242  0.368  0.123 | 0  0.026  0.133  0.017 | 0.5  0.511  0.572  0.516 | 0.651  0.149  0.333  0.104 |
| mRNALoc | Nucleus  Cytoplasm  ER | 0.391  0.466  **0.175** | 0.1461  -0.059  0.0621 | 0.565  0.452  0.546 | 0.710  0.480  0.095 |
| DeepLocRNA  (training from scratch) | Nucleus  Exosome  Cytosol  Ribosome  Membrane  ER | 0.7539  0.9961  0.0541  0.0692  0.0406  0 | 0.1592  0  0.0633  0.0159  0.1473  0 | 0.7429  0.7413  0.7404  0.7270  0.7446  0.6735 | 0.8561  0.9969  **0.3459**  0.5209  0.4226  0.2303 |
| DeepLocRNA  (instructive fine-tuning + weighting) | Nucleus  Exosome  Cytosol  Ribosome  Membrane  ER | **0.8341**  **0.9961**  0.2016  0.3860  **0.3001**  0.0702 | 0.3516  0  **0.1627**  0.2460  0.2569  **0.0474** | 0.7647  0.7537  0.7462  0.7564  0.7532  **0.7217** | 0.8681  0.9968  0.3383  0.5509  0.4337  **0.2836** |
| DeepLocRNA  (instructive fine-tuning) | Nucleus  Exosome  Cytosol  Ribosome  Membrane  ER | 0.8284  0.9960  **0.0921**  0.3382  **0.2677**  0 | **0.3653**  0  0.1156  0.2582  **0.2602**  0 | **0.7760**  **0.7633**  **0.7476**  0.7546  **0.7551**  0.6644 | **0.8764**  **0.9971**  0.3317  0.5475  0.4386  0.2295 |

**Supplementary table 1.** Comparative analysis of DeepRBPLoc and its counterparts using the segregated benchmark dataset. Bold numbers indicate the highest values observed across all comparisons within a given compartment.

| **RNA Species** | **Compartments** | **F1** | **MCC** | **AUROC** | **AUPRC** |
| --- | --- | --- | --- | --- | --- |
| mRNA | Nucleus  Exosome  Cytosol  Ribosome  Membrane  ER | **0.8252** \| 0.8019  **0.9961** \| 0.9960  **0.0109** \| 0  **0.3489** \| 0.2776  **0.2627** \| 0.0107  **0.0039** \| 0 | **0.3840** \| 0.3611  0 \| 0  **0.0270** \| 0.0024  **0.2678** \| 0.2101  **0.2560** \| 0.0532  **0.0137** \| 0 | **0.7752** \| 0.7654  **0.7598** \| 0.7325  **0.7435** \| 0.7169  **0.7607** \| 0.7539  **0.7551** \| 0.7416  **0.6655** \| 0.5966 | **0.8761** \| 0.8700  **0.9968** \| 0.9960  **0.3244** \| 0.2943  **0.5624** \| 0.5381  **0.4412** \| 0.4164  **0.2232** \| 0.1600 |
| miRNA | Nucleus  Exosome  Cytoplasm  Microvesicle  Mitochondrion | **0.8872** \| 0.7973  0.9149 \| **0.9431**  **0.0762** \| 0  0.9633 \| **0.9827**  0 \| 0 | **0.8524** \| 0.7499  0.2865 \| **0.4801**  **0.1057** \| 0.0056  0.8470 \| **0.9056**  0 \| 0 | **0.9760** \| 0.9028  0.9327 \| **0.9526**  **0.9008** \| 0.8008  0.9757 \| **0.9776**  0.5556 \| **0.7018** | **0.8987** \| 0.7988  0.9879 \| **0.9938**  **0.4808** \| 0.3750  0.9932 \| **0.9938**  0.0241 \| **0.2431** |
| lncRNA | Nucleus  Exosome  Cytosol  Membrane | **0.0965** \| 0.0223  0.9864 \| **0.9867**  0 \| 0  0 \| 0 | 0.0174 \| **0.0477**  **0 .0019** \| 0  0 \| 0  **0.0015** \| 0 | 0.5354 \| **0.5483**  0.6582 \| **0.6801**  **0.5872** \| 0.5723  0.5520 \| **0.5645** | 0.2701 \| **0.2724**  0.9863 \| **0.9872**  **0.1021** \| 0.0918  0.0461 \| **0.0483** |
| snoRNA | Nucleus  Exosome  Cytoplasm  Microvesicle | **0.1182** \| 0.0161  1 \| 1  0 \| 0  **0.9991** \| 0.9988 | **0.0993** \| 0.0437  0 \| 0  0 \| 0  0 \| 0 | **0.6541** \| 0.6340  / \| /  **0.6363** \| 0.5346  / \| / | **0. 3320** \| 0.2868  / \| /  **0.1878** \| 0.1075  / \| / |

**Supplementary table 2.** Performance of the unified model. The bold numbers represent the larger values when compared with the instructive fine-tuning model (left) and training from scratch model (right). The AUROC of exosome and microvesicle in snoRNA is nan because there are no false negative predictions calculated. The thresholds used for calculating F1 and MCC across all methods were 0.5.

| **AGGTA** | **UUUUU** | **TTCCG** | **GATGA** |
| --- | --- | --- | --- |
| NCBP2(612) | SUGP2(4924) | ILF3(1359) | NOL12(2117) |
| PPIG(1556) | FUBP3(4865) |  | TBRG4(1727) |
| SND1(1018) | YBX3(1336) |  | SDAD1(1227) |
| RBM22(2680) | U2AF1(3176) |  | SMNDC1(1080) |
| UCHL5(2182) | HLTF(4284) |  | WDR43(1374) |
| LSM11(954) | POLR2G(1954) |  |  |
| BUD13(2142) | DDX55(2646) |  |  |
| EIF3H(1819) | DDX6(1046) |  |  |
| CDC40(2275) | SLTM(721) |  |  |
| EIF3D(1322) | MATR3(4963) |  |  |
| TRA2A(1269) | TIAL1(2383) |  |  |
| AQR(3604) | DDX52(1765) |  |  |
|  | STAU2(2696) |  |  |
|  | FUS(706) |  |  |
|  | LARP4(3257) |  |  |
|  | AKAP1(1282) |  |  |
|  | SAFB(3316) |  |  |
|  | TIA1(4332) |  |  |
|  | PABPN1(1409) |  |  |
|  | EIF3D(2049) |  |  |

**Supplementary table 3.** 4 extracted motifs that were also found in RBPnet. The proteins displayed following the motifs are those that bind to these specific motifs. The numbers in parentheses represent the support count of these motifs in RNA-binding proteins (RBPs).

| **Species** | **Compart**  **ments** | **Nucleus** | **Microvesicle** | **Mitochondrion** | **Exosome** | **Cytosol** | **Cytoplasm** | **Ribosome** | **Membrane** | **ER** |
| --- | --- | --- | --- | --- | --- | --- | --- | --- | --- | --- |
|  | **lncRNA** | 836 | 1 | 1 | 3092 | 246 | 103 | 18 | 124 | 2 |
|  | **miRNA** | 472 | 1466 | 32 | 1605 | 0 | 234 | 1 | 0 | 0 |
| **Human** | **snoRNA** | 115 | 475 | 0 | 483 | 4 | 40 | 0 | 1 | 0 |
|  | **mRNA** | 11916 | 0 | 0 | 17136 | 2337 | 9646 | 5207 | 3231 | 1975 |
|  | **Sum** | **13352** | **1958** | **33** | **22335** | **2587** | **10026** | **5226** | **3356** | **1977** |
|  | **miRNA** | 62 | 8 | 95 | 301 | 0 | 7 | 0 | 0 | 0 |
| **Mouse** | **mRNA** | 2119 | 0 | 1 | 782 | 11 | 1491 | 2 | 1 | 8 |
|  | **Sum** | **2271** | **8** | **96** | **1116** | **13** | **1520** | **2** | **1** | **8** |

**Supplementary table 4.** Summary of the number of RNAs across nine subcellular compartments of humans and mice.

| **RNA species** | **Compartments** | **F1** | **MCC** | **AUROC** | **AUPRC** |
| --- | --- | --- | --- | --- | --- |
| mRNA | Nucleus  Exosome  Cytoplasm | **0.7704** \| 0.7694  **0.0370** \| 0.0248  **0.6700** \| 0.5965 | **0.5389** \| 0.5175  **0.1064** \| 0.0652  **0.5229** \| 0.4735 | **0.8414** \| 0.8263  **0.6268** \| 0.5975  **0.8405** \| 0.8202 | **0.8088** \| 0.7874  **0.3150** \| 0.2771  **0.7449** \| 0.7201 |
| miRNA | Nucleus  Exosome  Mitochondrion | 0 \| 0  0.9123 \| 0.9123  0 \| 0 | 0 \| 0  0 \| 0  0 \| 0 | **0.6317** \| 0.6279  **0.8125** \| 0.7843  **0.6980** \| 0.6937 | 0.2796 \| **0.3012**  **0.9504** \| 0.9423  0.5344 \| **0.5387** |

**Supplementary Table 5**. Performance of DeepLocRNA in mice in certain compartments. The bold numbers represent the larger values when compared with the instructive fine-tuning model (left) and training from scratch model (right).

| **Tools** | **Compartments** | **AUROC** | **AUPRC** | **MCC** |
| --- | --- | --- | --- | --- |
| DeepLncLoc | Nucleus  Exosome  Cytosol  Membrane | 0.5152  0.5002  0.4931  0.5 | 0.2619  0.9736  0.0701  0.0343 | 0.0266  0.0013  -0.0137  0 |
| LncLocator |  | 0.4913  0.5044  0.4904  0.5000 | 0.2534  0.9739  0.0701  0.0343 | -0.0263  0.0073  -0.0119  0 |
| iLoc-lncRNA |  | 0.5106  0.4965  0.5192  0.5000 | 0.2609  0.9735  0.0734  0.0343 | 0.0248  -0.0028  0.0206  0 |
| DeepRBPLoc  (training from scratch) |  | **0.5477**  0.5690  0.5839  0.5929 | 0.3000  0.9807  0.0959  0.0579 | 0.0156  0  0  0 |
| DeepRBPLoc  (instructive fine-tuning) |  | 0.5443  **0.5832**  **0.5932**  **0.5938** | **0.3027**  **0.9815**  **0.1004**  **0.0658** | **0.0156**  0  0  0 |

**Supplementary Table 6**. Benchmarking of DeepLocRNA in the prediction of lncRNA across four compartments.

**Reference**

[1. Wang, D.](https://www.zotero.org/google-docs/?rf4HWk) *[et al.](https://www.zotero.org/google-docs/?rf4HWk)* [DM3Loc: multi-label mRNA subcellular localisation prediction and analysis based on multi-head self-attention mechanism.](https://www.zotero.org/google-docs/?rf4HWk) *[Nucleic Acids Res.](https://www.zotero.org/google-docs/?rf4HWk)* **[49](https://www.zotero.org/google-docs/?rf4HWk)**[, e46–e46 (2021).](https://www.zotero.org/google-docs/?rf4HWk)

[2. Fu, L., Niu, B., Zhu, Z., Wu, S. & Li, W. CD-HIT: accelerated for clustering the next-generation sequencing data.](https://www.zotero.org/google-docs/?rf4HWk) *[Bioinformatics](https://www.zotero.org/google-docs/?rf4HWk)* **[28](https://www.zotero.org/google-docs/?rf4HWk)**[, 3150–3152 (2012).](https://www.zotero.org/google-docs/?rf4HWk)

[3. Zhang, Z.-Y.](https://www.zotero.org/google-docs/?rf4HWk) *[et al.](https://www.zotero.org/google-docs/?rf4HWk)* [iLoc-miRNA: extracellular/intracellular miRNA prediction using deep BiLSTM with attention mechanism.](https://www.zotero.org/google-docs/?rf4HWk) *[Brief. Bioinform.](https://www.zotero.org/google-docs/?rf4HWk)* **[23](https://www.zotero.org/google-docs/?rf4HWk)**[, bbac395 (2022).](https://www.zotero.org/google-docs/?rf4HWk)

[4. Wang, J., Horlacher, M., Cheng, L. & Winther, O. RNA trafficking and subcellular localisation—a review of mechanisms, experimental and predictive methodologies.](https://www.zotero.org/google-docs/?rf4HWk) *[Brief. Bioinform.](https://www.zotero.org/google-docs/?rf4HWk)* [bbad249 (2023) doi:10.1093/bib/bbad249.](https://www.zotero.org/google-docs/?rf4HWk)

[5. Horlacher, M.](https://www.zotero.org/google-docs/?rf4HWk) *[et al.](https://www.zotero.org/google-docs/?rf4HWk)* [Towards in silico CLIP-seq: predicting protein-RNA interaction via sequence-to-signal learning.](https://www.zotero.org/google-docs/?rf4HWk) *[Genome Biol.](https://www.zotero.org/google-docs/?rf4HWk)* **[24](https://www.zotero.org/google-docs/?rf4HWk)**[, 180 (2023).](https://www.zotero.org/google-docs/?rf4HWk)

[6. Van Nostrand, E. L.](https://www.zotero.org/google-docs/?rf4HWk) *[et al.](https://www.zotero.org/google-docs/?rf4HWk)* [A large-scale binding and functional map of human RNA-binding proteins.](https://www.zotero.org/google-docs/?rf4HWk) *[Nature](https://www.zotero.org/google-docs/?rf4HWk)* **[583](https://www.zotero.org/google-docs/?rf4HWk)**[, 711–719 (2020).](https://www.zotero.org/google-docs/?rf4HWk)

7 Bahdanau, D., Cho, K. & Bengio, Y. Neural Machine Translation by Jointly Learning to Align and Translate. Preprint at http://arxiv.org/abs/1409.0473 (2016).
